# Insights into the biogenesis and potential functions of exonic circular RNA

**DOI:** 10.1038/s41598-018-37037-0

**Published:** 2019-02-14

**Authors:** Chikako Ragan, Gregory J. Goodall, Nikolay E. Shirokikh, Thomas Preiss

**Affiliations:** 1 EMBL–Australia Collaborating Group, Department of Genome Sciences, The John Curtin School of Medical Research, The Australian National University, Canberra, ACT 2601 Australia; 2 Centre for Cancer Biology, University of South Australia and SA Pathology, Adelaide, SA 5000 Australia; 3 Discipline of Medicine, The University of Adelaide, Adelaide, SA 5005 Australia; 4 School of Molecular and Biomedical Science, The University of Adelaide, Adelaide, SA 5005 Australia; 5 Victor Chang Cardiac Research Institute, Darlinghurst, NSW 2010 Australia

## Abstract

Circular RNAs (circRNAs) exhibit unique properties due to their covalently closed nature. Models of circRNAs synthesis and function are emerging but much remains undefined about this surprisingly prevalent class of RNA. Here, we identified exonic circRNAs from human and mouse RNA-sequencing datasets, documenting multiple new examples. Addressing function, we found that many circRNAs co-sediment with ribosomes, indicative of their translation potential. By contrast, circRNAs with potential to act as microRNA sponges were scarce, with some support for a collective sponge function by groups of circRNAs. Addressing circRNA biogenesis, we delineated several features commonly associated with circRNA occurrence. CircRNA-producing genes tend to be longer and to contain more exons than average. Back-splice acceptor exons are strongly enriched at ordinal position 2 within genes, and circRNAs typically have a short exon span with two exons being the most prevalent. The flanking introns either side of circRNA loci are exceptionally long. Of note also, single-exon circRNAs derive from unusually long exons while multi-exon circRNAs are mostly generated from exons of regular length. These findings independently validate and extend similar observations made in a number of prior studies. Furthermore, we analysed high-resolution RNA polymerase II occupancy data from two separate human cell lines to reveal distinctive transcription dynamics at circRNA-producing genes. Specifically, RNA polymerase II traverses the introns of these genes at above average speed concomitant with an accentuated slow-down at exons. Collectively, these features indicate how a perturbed balance between transcription and linear splicing creates important preconditions for circRNA production. We speculate that these preconditions need to be in place so that looping interactions between flanking introns can promote back-splicing to raise circRNA production to appreciable levels.

## Introduction

Covalently closed RNA molecules exist across all branches of life^1–6^. Notable examples are viroids and intermediates of certain RNA processing reactions, including circularised forms of group I and II introns^1^. The products of eukaryotic spliceosomal action can also be circular^1^. First, intron lariats can sometimes escape debranching to form stable circular intronic (ci)RNA. Second, the spliceosome can also fuse exons out of their linear sequence. This ‘back-splicing’ phenomenon generates exonic or mixed exonic-intronic circular (circ)RNAs^1^. A small number of back-spliced circRNAs were already characterised decades ago and thought to be rare oddities^4,7,8^, however, high-throughput RNA sequencing (RNA-seq) has since revealed the existence of thousands more examples in a range of eukaryotic species^9–16^.

CircRNAs vary widely in abundance and their levels often are not directly related to those of the corresponding linear (mRNA) counterparts. The molecular and cellular roles of the vast majority of circRNAs are unknown^1–3,10,17,18^, although some are conserved across species, suggesting their importance^9,11,13–16,19,20^. Cellular localisation can be an indicator of potential function. For example, the ciRNAs as well as circRNAs with retained introns mainly accumulate in the nucleus and are thought to regulate transcription^2,21–23^. By contrast, purely exonic circRNAs are mostly exported to (or occur in) the cytoplasm^2,9,13^, where they might be involved in post-transcriptional gene regulation^9^. Indeed, the murine circular RNA *Sry* (Sex-determining region Y) and a human circRNA running antisense to the *CDR1* (Cerebellar Degeneration-Related protein 1; CDR1as) locus were both found to act as micro (mi)RNA sponges. Each carries multiple binding sites for a specific miRNA, miR-138 and miR-7 respectively, and their presence in cells mitigates the repression of the corresponding target messenger RNAs (mRNAs)^12,14^. Although some additional examples of potent miRNA sponging by single circRNAs have since been reported^24–29^, this is thought to be a rare occurrence since circRNAs typically do not exhibit such an accumulation of miRNA binding sites^15^.

Another emerging circRNA function in the cytoplasm is as a template for protein synthesis^2,30,31^. This is not an implausible premise as artificial circRNAs have been shown to be translatable both *in vitro*^32^ and *in vivo*^33^. Indeed, the endogenous human circ-*ZNF609* harbours an open reading frame that runs across the back-splicing junction and was shown to be translated at a low level^34^. Criteria such as ribosome footprints and mass spectrometry-based detection of peptides that span the back-splice junction were used to screen for circRNA translation in fruit flies^35^ and human cells^36^, although detection limits meant that only a modest number of cases were firmly identified (*e*.*g*. 19 additional human examples in ref.^36^). Translation of circRNA must involve some form of internal ribosome entry and a mechanism involving recruitment of translation initiation complexes by the *N*^6^-methyladenosine (m^6^A) RNA modification has emerged as a front runner^36^. Notwithstanding these interesting examples, it remains to be seen how commonly circRNAs engage with ribosomes and if the levels and features of the encoded polypeptides are such that they can typically serve a physiological purpose.

The mechanism of circRNA production by back-splicing is also an active area of research. Most known circRNAs derive from internal exons of protein-coding genes and they require both 5′ and 3′ canonical splice-site signals for their generation^37^. CircRNAs typically contain a relatively small number of exons^38^, with the second exon of the cognate pre-mRNA predominantly acting as the back-splice acceptor^9,39^. Single-exon circRNAs were reported to contain longer exons^38^. These features could relate to either circRNA biogenesis or potential function. Also noted were unusually long introns on both flanks of the circRNA producing region^9,13,24^, and an enrichment of reverse complementary motifs (RCMs; *e*.*g*. diverging Alu elements in the context of the human genome) within these introns^38,40–42^. RCMs are thought to form loops in pre-mRNA by complementary interactions to facilitate back-splicing^13,41^; these RCM interactions can further be modulated by RNA-binding proteins (RBPs)^43^ and RNA editing^41^ to inhibit circRNA biogenesis. Conversely, the RBP Quaking (QKI) has been shown to favour back-splicing to yield hundreds of circRNAs in a manner similar to RCMs, by binding to flanking introns to promote looping^44^. Fruit fly Muscleblind (Mbl) autoregulates its own production, by binding to flanking introns of its own pre-mRNA and diverting synthesis into circRNA^45^. Exon skipping and lariat bridging are also among the possible pro-back-splicing factors^4,46^.

Despite the above evidence, circRNA biogenesis by flanking intron looping could not work if splicing was strictly co-transcriptional. Thus, circRNA producing regions must exhibit some features to ensure that the upstream splice acceptor is still available by the time the downstream donor gets transcribed. First, the unusually long introns that flank circRNAs could underlie the observed reduction in efficiency of linear splicing for the exons involved^45^. Second, circRNA-producing genes were found to be transcribed at a faster-than-average rate^47^ and fruit fly mutants with a lower RNA polymerase II (Pol II) elongation rate had depleted circRNA levels^45^. Third, in fly mutants with depleted spliceosome levels, circRNAs can become the preferred gene output^48^. Taken together with the fact that Pol II elongation rate and efficiency of linear splicing are inversely correlated^49^, these observations indicate that back-splicing is dependent on mechanisms that delay or otherwise compromise linear splicing at circRNA loci.

Here, we used existing deep RNA-seq data^50,51^ to computationally identify 794 human and 1,541 mouse circRNAs that are generated from protein-coding genes by a back-splicing mechanism. We assessed potential functions of the human circRNAs. Based on co-sedimentation with poly(ribo)somal complexes we shortlist 177 of the human circRNAs as candidates for translation. We found no convincing evidence for individual circRNAs acting as miRNA sponges, but there was a potential for groups of circRNAs acting as ‘collective sponges’ for a small number of miRNAs including the let-7 and miR15/16 miRNA families. We noted that the human and mouse circRNAs identified here preferentially derive from multi-exon genes. CircRNA gene loci are typically flanked by long introns, incorporate exons that emerge early during gene transcription and give rise to circRNAs with a short exon span. Analysis of genome-wide Pol II density maps^52^ further revealed that human circRNA-producing genes are transcribed faster than average but feature accentuated exon-intron Pol II speed differences and pausing at intron-exon boundaries.

## Results

### Computational prediction of human and mouse circRNAs

**Figure 1:**
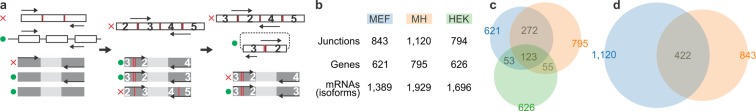
Detection of human and mouse circRNAs in paired-end RNA-seq data^50,51^. (**a**) Schematic of the computational pipeline that discards (red ) read pairs that map to the linear transcriptome and identifies pairs where one read maps to a back-spliced junction while the other read maps within the exon span of the putative circRNA (green ).  Denotes canonical linear-spliced junction,  denotes back-spliced junction. Numbers indicate ‘linear’ ordinal exon position in a gene. (**b**) Numbers of predicted circRNAs (‘junctions’) with ≥0.1 junction per million of reads (JPM), circRNA-producing loci (‘genes’), and related RefSeq mRNA isoforms in MEF cells (blue), mouse heart (MH; red) and HEK 293 cells (green). (**c**) Overlap of circRNA-producing genes between all three sources. (**d**) Overlap of circRNAs identified in the different mouse sources.

We used available ribosomal RNA-depleted, but not poly(A)^+^-selected, paired-end RNA-seq data^50,51^ (~30–200 million read pairs per sample, see ‘RNA sequencing data’ subsection in the Methods) for identifying circRNAs using a stringent computational pipeline, similar to one described previously^9^. Briefly, we first removed read pairs that mapped to the reference genome based on linear transcript structures (as annotated in GENCODE). To identify circRNAs, we then collected reads that uniquely mapped to back-spliced exon junctions (based on RefSeq mRNAs) and required that the second read of the pair mapped to sequences located inside of the putative circle (Fig. 1a).

We selected a dataset from HEK 293 human embryonic kidney cells as it included multiple RNA samples that were fractionated based on ribosome association prior to sequencing (see below)^50^. CircRNAs are thought to be important in development and cardiac function^53–56^ and thus we also included data for total RNA from mouse embryonic fibroblasts (MEFs) and adult mouse hearts^51^. After implementing criteria for moderate circRNA abundance^38^ (generally ≥0.1 junction per million of mapped reads (JPM) across each cell type, see ‘Verification of circRNAs and normalisation of the read counts’ subsection in the Methods), we predict 794 unique circRNAs in HEK 293 cells, derived from 626 protein-coding genes and potentially related to 1,696 different linear mRNA isoforms (MEFs: 843 circRNAs, 621 genes, 1,389 mRNA isoforms; mouse heart: 1,120 circRNAs, 795 genes, 1,929 mRNA isoforms; Fig. 1b; complete list in Supplementary Table S1). ~64% (395 of 621) of the circRNA-producing genes (Fig. 1c) and ~50% (422 of 843) of the predicted circRNAs (Fig. 1d) overlap between MEFs and mouse hearts. ~37% (231 of 626) of the circRNA-producing genes were conserved between human and mouse (Fig. 1c). This is comparable to previous studies that had shown an overlap of ~350 circRNA-producing genes between human and mouse cells^15^. Further, we find a ~46% overlap (111 of 239) with a previously reported set of circRNAs for HEK 293 cells^14^ and a ~32% overlap (810 of 2,561) with either one or both published circRNA predictions from mouse hearts^57,58^ (Supplementary Fig. S1). Given the many differences in the depth of source data as well as the scope and stringency of circRNA prediction^59^, this represents a respectable overlap of circRNAs across different studies. We conclude that our circRNA prediction approach is reasonably balanced, as it independently and substantially confirms previously reported circRNAs, while detecting multiple novel human and mouse circRNAs.

### Assessing circRNA potential as miRNA sponges

To conservatively annotate potential miRNA binding sites within our set of HEK 293 circRNAs, we used Argonaute (AGO) 1–4 binding sites identified by Cross-Linking ImmunoPrecipitation sequencing (CLIP-seq) in the same cell line^60,61^ combined with detection of underlying miRNA seed matches (see ‘Detection of RBP binding sites’ and ‘Detection of miRNA binding sites’ subsections in the Methods). This showed a mild underrepresentation of AGO2 footprints in circRNAs compared to exclusively linear exons, following a tendency seen for several other RBPs (Supplementary Fig. S2).

~12% (97 of 794) of circRNAs contain one or more miRNA target sites predicted by these criteria (Supplementary Table S2). Most of these had only a single short AGO footprint region, which, even though it typically covered multiple potential seed matches overlapping each other, precluded potent miRNA sponging. We found one example with some potential in a circRNA derived from the *CPSF6* gene. It harbours extended regions of AGO footprint density, which contain two sets of twelve well-dispersed seed matches, one for GGUGGA and one for UGGAGG seeds. However, apart from miR-4443, which has roles in carcinogenesis and immune response^62–65^, functions of the other identified target miRNAs are not characterised. The well-known miRNA sponges *Sry* and *CDR1as*^12,14^ were not represented in our circRNA detection data. We also searched for occurrence of particular seed matches across multiple circRNAs and this gave a number of hits, *e*.*g*. thirteen seed match types that were present eight times or more (Supplementary Table S2). Interestingly, this included eight matches to the GAGGUA seed representing members of the let-7 family and ten matches to the AGCAGC seed representing the miR-15/16 family of miRNAs, both highly conserved miRNA families with prominent roles in development, cellular homeostasis and cancer^66,67^. Taken together, we found some indication for ‘collective’ sponge action by groups of human circRNAs.

### Identifying circRNAs with potential for translation

**Figure 2:**
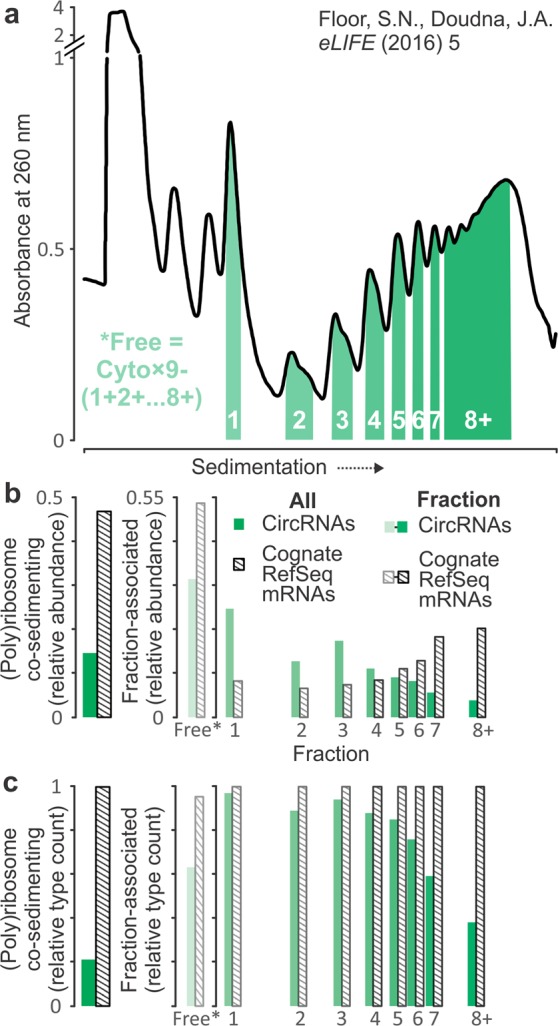
Selection of human translation candidate (tc-) circRNAs from Transcript Isoform in Polysomes sequencing (TrIP-seq) data. (**a**) UV absorbance profile after separating cycloheximide-treated cytoplasmic HEK 293 cell extract, showing how RNA co-sedimenting with different (poly)ribosomal fractions was collected and sequenced separately. Figure reproduced with modifications, and non-processed sequencing data taken, from the original publication^50^. (**b**) Relative abundance of circRNA (JPM; green) and cognate linear mRNA (RPM; striped) overall for the entire set of HEK circRNAs (left) and across (poly)ribosome co-sedimenting and non-associated (free*; calculated) RNA (right). (**c**) Same as (**b**), but comparing the presence of circRNAs (counts of unique back-spliced junctions) to that of the corresponding cognate linear mRNAs. *’Free’ denotes RNA pool not associated with ribosomes calculated from counts in ribosomal fractions and total cellular RNA as indicated in panel (a) (also see ‘Detection of circRNAs in ribosome sedimentation profiles’ subsection in the Methods for details).

The HEK 293 cell data were originally used to identify mRNA isoform-specific translational control using an approach termed Transcript Isoform in Polysomes sequencing (TrIP-seq)^50^. For this purpose, cytosolic extracts were first separated by ultracentrifugation in sucrose gradients (Fig. 2a). RNA fractions were obtained based on co-sedimentation with mono- and poly-ribosomal complexes and sequenced alongside unfractionated cytosolic RNA. Translation of both, linear and circular RNA, is indicated by their sedimentation into the polysomal region and extrapolated ribosome density can be used as a measure of translation efficiency. We found ~15% of all circRNA read counts in the ribosomal fractions (Fig. 2b, left; see Supplementary Fig. S3o for the corresponding read frequencies from all mRNA-producing RefSeq genes). We detected 177 of the circRNAs with an average JPM threshold ≥0.1 across the eight ribosomal fractions (representing ~22% (177/794) of circRNAs; Fig. 2c, left) and thus identified them as translation candidate (tc-circRNAs) (Supplementary Table S3). Based on both, the number of circRNAs or read counts, tc-circRNAs are most strongly represented in the monosomal to tetrasomal fractions, with a marked decline towards the octasome-plus fraction (Fig. 2b,c, right; see Supplementary Fig. S3o,p for the corresponding counts from all mRNA-producing RefSeq genes). By contrast, all cognate linear mRNAs are detectable across all fractions and they are more abundant in the fast-sedimenting (mostly, hepta- and octasome-plus) fractions. These differences between tc-circRNAs and cognate linear mRNAs are in part explained by the far greater sensitivity of linear mRNA detection combined with their often-higher abundance. But they also match expectations that (a) not all circRNAs are likely to interact with ribosomes, and (b) circRNAs can accommodate only a few ribosomes concurrently, due to their shorter average length (411 nt for tc-circRNAs, compared to 4,761 nt for their cognate linear mRNAs).

All circRNAs described here contain features typical of translated sequences, as they consist of exons from protein-coding genes. Thus, we focussed on detecting enrichment of specific features in tc-circRNAs relative to those circRNAs that were absent from ribosome fractions. We found an over-representation of relatively short ORFs beginning with near-cognate CUG and GUG codons (Supplementary Fig. S3l and n), but no other features such as overall ORF length or presence of m^6^A sites (Supplementary Fig. S3) exhibited statistically significant differences in this comparison.

Overall, we show here that TrIP-seq can be used for sensitive detection of potential circRNA translation and we provide a set of 177 human tc-circRNAs as candidates for such a function. The well-characterised circ-*ZNF609*^34^ was not detected in our data. Of interest, Yang *et al*. had used a combination of sedimentation, RNase R digestion and RNA-seq to identify a set of 250 potentially polysome-associated circRNAs from HeLa human cervical cancer cells; translation of 19 circRNAs was furthermore evidenced by identification of back-splice junction-spanning peptides in mass spectrometry data across different cell types and datasets^36^. We find 53 of these 250 candidates in our overall HEK 293 circRNA identification list, and 32 were among our tc-circRNA set (one overlapped with the set of 19 confirmed by mass-spectrometry). Given the differences in source material, sequencing strategy and criteria chosen as indicative for translation, this represents a reasonable concordance between the different studies.

### CircRNA-producing genes have a splice-isoform-generating capacity comparable to genes of similar structure

**Figure 3:**
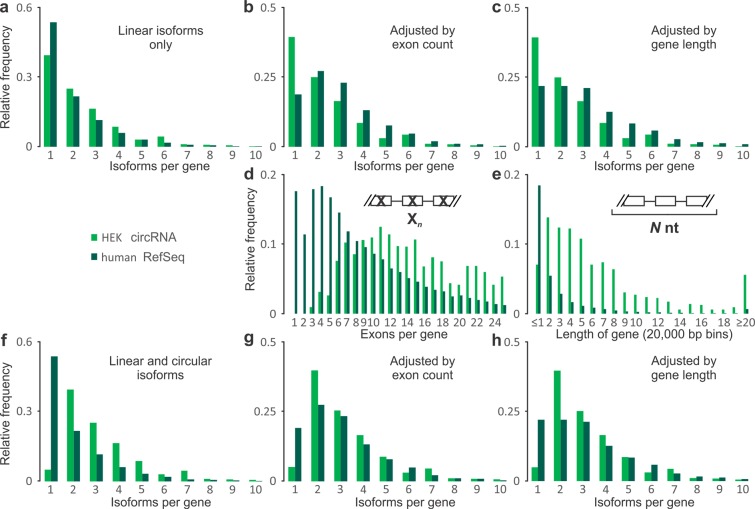
Linear isoform diversity of human HEK 293 cell circRNA-producing genes. (**a**) mRNA isoform frequency of circRNA-producing genes (green) compared to all human RefSeq genes (dark green). (**b**) Same as (**a**), but compared to exon-count-adjusted genes (dark green). (**c**) Same as (**b**), but compared to gene-length-adjusted genes (dark green). (**d**) Exon counts in circRNA-producing genes compared to RefSeq genes. (**e**) Same as (**d**) but for gene lengths (binned in 20,000 bp steps). (**f**–**h**) As (**a**–**c**) but circRNAs are counted as additional transcript isoforms for each gene. Designations and abbreviations as in Fig. 1. The distributions of data in (**a**,**d**–**f** and **h**) are significantly different between circRNA-producing and reference genes (P-value < 0.01, Mann-Whitney U test).

Next, we inspected the overall structure of circRNA-producing genes. We investigated whether circRNA production is correlated with an increase in annotated alternative splicing variants. Although not all circRNAs were descendant from genes that produce alternatively spliced variants, we indeed found in both, human and mouse, a preference for back-splicing occurrence in genes that also produce multiple isoforms of linear mRNA (Fig. 3a; see Supplementary Fig. S4a for data from all three sample types). However, circRNA-synthetizing genes on average have more than twice the number of exons per gene compared to the RefSeq gene set (Figs 3d and S4d) and their average gene lengths are also ~1.6 to ~1.8 times higher (Figs 3e and S4e). To correct for this, we generated exon-count-adjusted and gene-length-adjusted reference datasets in which RefSeq genes were randomly selected to match with equivalent average exon numbers or lengths of circRNA-producing genes (see ‘Custom reference datasets’ subsection in the Methods). This like-for-like comparison showed that control genes featured more linear isoforms per gene than circRNA-producing genes (Figs 3b,c and S4b,c). When circRNAs and linear mRNAs isoforms are counted together, circRNA-producing genes come out slightly but significantly ahead (Figs 3g,h and S4g,h). Thus, there is a bias towards circRNA-production from longer, multi-exon genes where they, however, largely form part of the expected multeity of transcript isoforms.

### CircRNAs typically have a short exon span and include exons that emerge early during transcription

**Figure 4:**
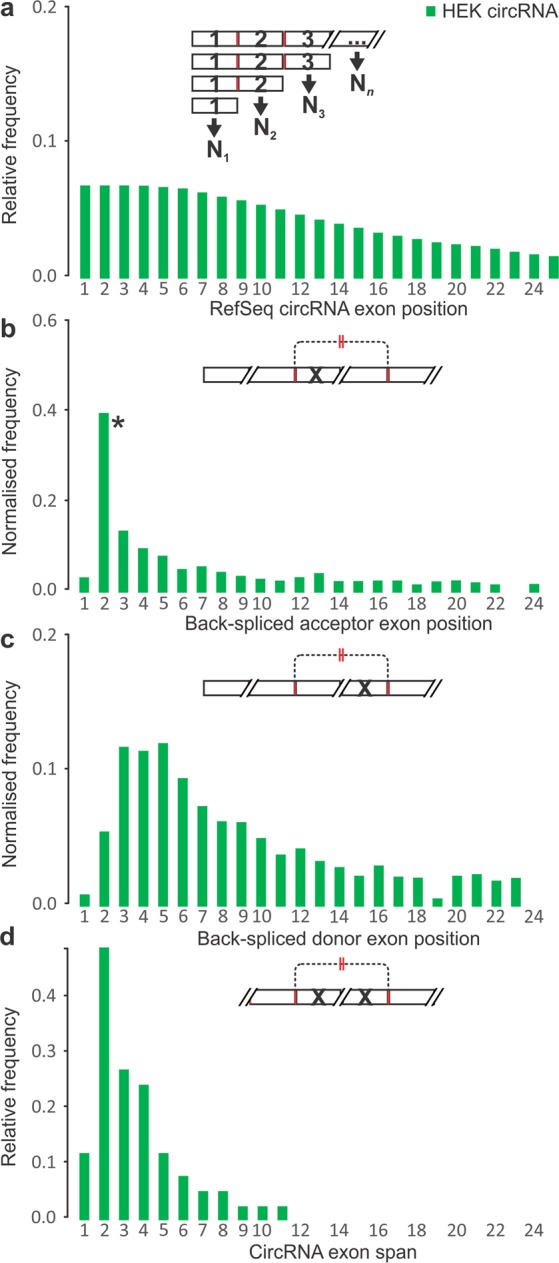
Exonic features at human HEK 293 cell circRNA-producing gene loci. (**a**) Relative frequencies of exons by ordinal position N along circRNA-producing genes. Frequency of back-splice (**b**) acceptor and (**c**) donor exon at each position after normalisation to exon frequency as shown in (**a**). *CircRNA acceptor exons were over-represented in the second position of circRNA-producing genes compared to other exon positions (P-value < 0.01, Mann-Whitney U test). (**d**) CircRNA span in exons. Note: where applicable, each RefSeq annotated mRNA was considered as a potential cognate linear isoform for a given circRNA. This ‘inclusive’ approach was used to avoid bias; however, an unavoidable consequence is a low frequency of erroneous acceptor and donor assignment to exon position 1. Designations and abbreviations are as in Fig. 1.

We determined the preferred positions of acceptor and donor exons within circRNA-producing genes (Fig. 4 shows results for human HEK 293 cells; see Supplementary Fig. S5 for a juxtaposition of human and mouse data). To do this, we corrected exon frequency for a decline in mRNA prevalence as exon numbers increase (Figs 4a and S5c,f). There was a significant and pronounced preference for the second transcribed exon acting as back-splice acceptor for circRNA generation (Fig. 4b) and this was seen irrespective of normalisation for exon frequency and across all three, human and mouse circRNA sets (Supplementary Fig. S5a,d,g). The distribution of donor exons showed a broad peak around exon positions 3–5, again in both human and mouse (Figs 4c and S5b,e,h). Consistent with the above, the great majority of human and mouse circRNA had a span of 1–4 exons, with a pronounced peak at two exons (Figs 4d and S6; a caveat is that our analyses did not test for any potential skipping of internal exons).

Thus, circRNAs arise from a wide range of exon positions and combinations. Nevertheless, and irrespective of the source data, we show that circRNAs exhibit a strong preference for inclusion of the second linearly transcribed exon and a span of around two exons. These findings independently confirm and extend previous reports, *e*.*g*. the second exon was found to be the preferred back-splice acceptor in human leukocytes^9^, and that circRNA span was seen to peak at two exons in H9 human embryonic stem cells^38^.

### CircRNA-generating exons tend to be of untypical lengths

**Figure 5:**
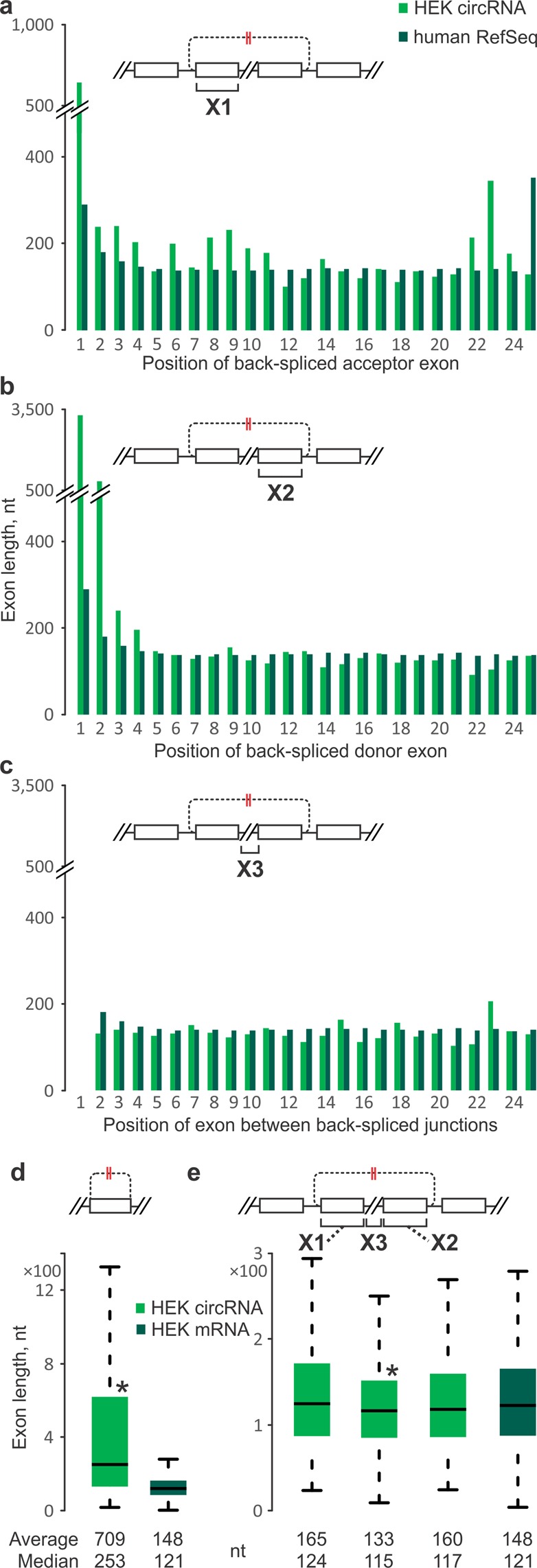
Exon lengths at human HEK 293 cell circRNA loci. Exons from circRNA-producing genes (green) are compared to RefSeq (**a**–**c**) or HEK-specific RefSeq (**d**,**e**) averages in the corresponding linear ordinal positions (dark green). (**a**) Average acceptor exon lengths. (**b**) Same as (**a**), but for donor exons. (**c**) Same as (**a**), but for circRNA-internal exons. (**d**) Single-exon circRNAs (green) compared to the internal exon lengths of HEK-specific RefSeq mRNAs (dark green). (**e**) Same as (**d**), but for acceptor (X1), donor (X2) and internal (X3) exons of multi-exon circRNAs. (**d**,**e**) Asterisks denote significantly different exon lengths between the circRNA-producing genes and HEK-specific RefSeq mRNAs (P-values < 0.01, Mann-Whitney U test). Designations and abbreviations are as in Fig. 1. Note: introns located between exons 1 and 2 of each RefSeq-annotated transcript were denoted as intron 1. Where applicable, all RefSeq annotated mRNAs (linear isoforms) overlapped with a circRNA were considered as potential cognate linear isoforms for a given circRNA.

Next, we analysed the lengths of exons that give rise to circRNAs. Differences in circRNA exon lengths were hard to discern when compared to internal exons in an ordinal-position resolved manner, of all RefSeq genes or just those expressed in the cell/tissue type (see Figs 5a and S7a,d for acceptor, Figs 5b and S7b,d for donor, and Figs 5c and S7c,f for internal exons; see ‘Custom reference datasets’ subsection in the Methods). First and last exons of mammalian genes tend to be long and also vary in size, while internal exons are of a more uniform length irrespective of their ordinal position^68^. Indeed, internal exons derived from our sets of expressed human and mouse mRNAs gave average lengths of 148–149 nt and a median of 121 nt (Tables 1 and S4), which is in-line with similar published analyses and with expectations that, at the DNA level, internal exons are considered to feature a well-positioned single nucleosome with 147 bp of DNA wrapped around the histone core^49,69–71^. Compared to those figures, single-exon circRNAs were significantly longer (averages of 404–709 nt and medians of 169–253 nt, depending on source; Fig. 5d; Tables 1 and S4), consistent with previous observations in H9 human embryonic stem cells^38^. Multi-exon circRNAs did not show such strong trends, albeit that acceptor and donor exons tended to be marginally longer, while internal exons tended to be shorter than the reference (Fig. 5e; Tables 1 and S4). These differences reached statistical significance only for the circRNA-internal exons being shorter (Tables 1 and S4). Overall, this suggests that exons involved in circRNA generation tend to deviate from the typical gene-internal exon length; this effect is subtle for multi-exon circRNAs but pronounced for the unusually long single-exon circRNAs.

**Table 1:** Lengths values^a^ for exons and introns of circRNAs compared to the values for HEK-specific gene set.

	Genes^b^	Exons						Introns					
		Acceptor^c^		Internal		Donor		Acceptor		Internal		Donor	
HEK 293	RefSeq	N/A		148.3		N/A		N/A		5,825.1		N/A	
				121						1,541			
	Single-exon circRNAs	708.9	**<2.2E-16**	N/A		708.9	**<2.2E-16**	19,985.0	**<2.2E-16**	N/A		17,530.5	**<2.2E-16**
		253	**<2.2E-16**			253	**<2.2E-16**	8,413	**<2.2E-16**			5,898	**1.1E-14**
	Multi-exon circRNAs	164.7	**1.1E-1**	132.5	**4.8E-7**	159.7	**7.6E-2**	20,627.6	**<2.2E-16**	4,890.8	**<2.2E-16**	20,433.4	**<2.2E-16**
		124	**4.4E-2**	115	**5.8E-9**	117	**5.8E-3**	11,761	**<2.2E-16**	2,394	**<2.2E-16**	11,216	**<2.2E-16**

^a^Average (top) and median (bottom) length values in nucleotides. ^b^Cell-specific gene set included RefSeq genes which sequences were detected in the respective HEK 293 RNA-seq data (see ‘Custom reference datasets’ subsection of the Methods for details). ^c^Bold numbers show P-values of Mann-Whitney U test between the distributions of relevant features of circRNAs and the internal exons (the first and last exons were removed) and introns of expressed RefSeq genes.

### CircRNA back-splicing acceptor and donor sites are typically flanked by long introns

**Figure 6:**
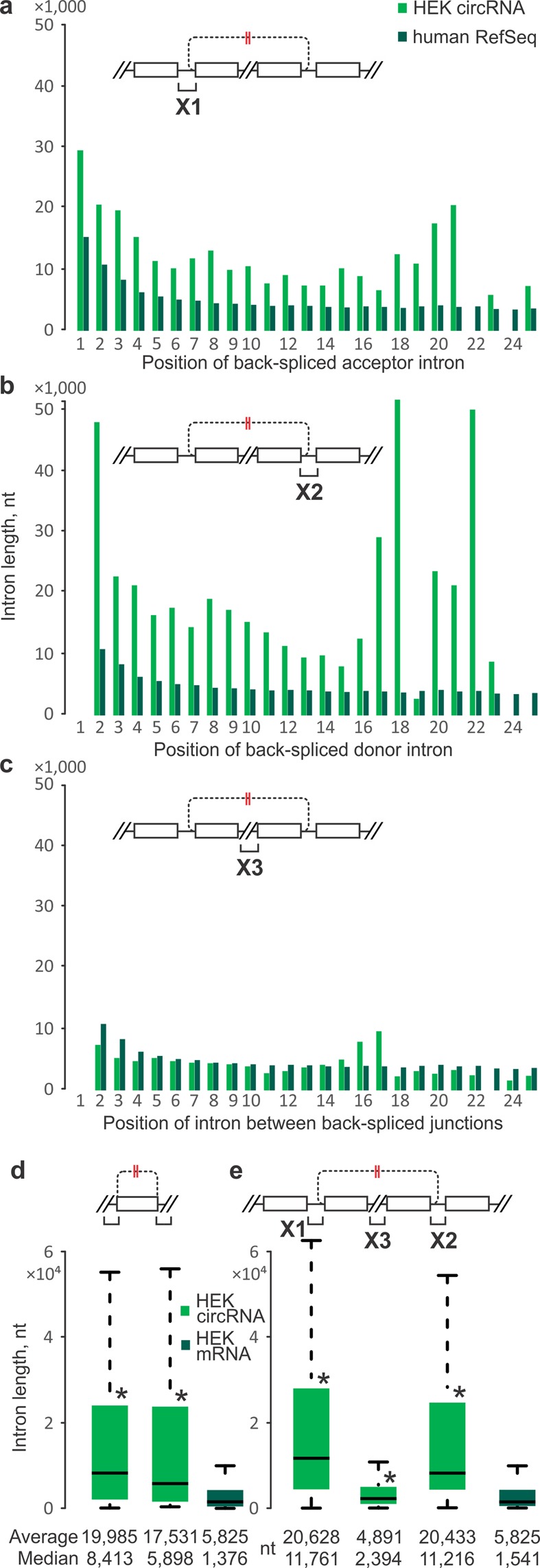
Intron lengths at human HEK 293 cell circRNA loci. Introns from circRNA-producing genes (green) are compared to RefSeq averages in the corresponding linear ordinal positions (dark green). (**a**) Average intron lengths at the upstream flank of back-spliced acceptor exons. (**b**) Same as (**a**), but for introns at the downstream flank of back-spliced donor exons. (**a**,**b**) Acceptor and donor introns of circRNA-producing genes are much longer than RefSeq genes in same ordinal positions and overall lengths of acceptor and donor introns were significantly longer than introns of RefSeq genes (measured by P-value < 0.01, Mann-Whitney U test). (**c**) Same as (**a**), but for circRNA-internal introns. (**d**) All single-exon circRNAs acceptor (left) and donor (right) intron lengths values (green) compared to the intron lengths of HEK-specific RefSeq mRNAs (dark green). (**e**) Same as (**d**), but for acceptor (X1), donor (X2) and internal (X3) introns of multi-exon circRNAs. (**d**,**e**) Asterisks denote significantly different intron lengths between the circRNA-producing genes and HEK-specific RefSeq mRNAs (P-values < 0.01, Mann-Whitney U test). Designations and abbreviations are as in Fig. 1. Note: introns located between exons 1 and 2 of each RefSeq-annotated transcript were denoted as intron 1. Where applicable, all RefSeq annotated mRNAs (linear isoforms) overlapped with a circRNA were considered as potential cognate linear isoforms for a given circRNA.

Next, we analysed the lengths of introns in circRNA-producing genes. Interestingly, mammalian genes show clear intron length trends with ordinal position. First and other early position introns tend to be longer than those in subsequent positions (Figs 6 and S8)^68^. Introns flanking circRNA junctions followed this trend but were still much longer than introns of all RefSeq genes irrespective of ordinal position within the gene. This was seen for both introns at the upstream flank of acceptor exons (Figs 6a and S8a,d) and at the downstream flank of donor exons (Figs 6b and S8b,d). By contrast, circRNA-internal introns were largely of a length commensurate with their ordinal position (Figs 6c and S8c,f). Flanking introns of both single-exon and multi-exon circRNAs were substantially longer than reference introns (Tables 1 and S4). We saw no pronounced length differences between upstream and downstream flanking introns, contrasting with expectations based on their difference in distribution across ordinal positions. These results independently confirm and extend previous findings with diverse *Drosophila* species’ cells^24^, as well as human fibroblasts^13^, H9 embryonic stem cells^38^ and lymphoblastic leukaemia diagnostic bone marrow samples^9^, although the latter study had reported that introns on the upstream flank were longer than those on the downstream flank.

### Accentuated differences in transcription speed between introns and exons are features of circRNA-producing genes

Finally, we took advantage of available ‘Native Elongating Transcript sequencing’ (NET-seq) data from HEK 293 and HeLa cells^52^ to analyse transcription dynamics at circRNA-producing genes. NET-seq globally maps 3′-ends of nascent transcribed RNA at nucleotide resolution. Thus, NET-seq signals along protein-coding genes represent a composite picture of gene transcription frequency and RNA polymerase II (Pol II) dwell time, an inverse of elongation speed. To eliminate the effects of gene transcription frequency, we first established a set of RefSeq genes that matches our circRNA genes in overall expression level (see ‘Custom reference datasets’ subsection in the Methods). Using the NET-seq signal intensity as a surrogate measurement for inverse Pol II elongation speed in this way we then performed a series of comparisons between those two gene groups (Table 2).

**Table 2:** NET-seq signal values (Pol II/nt)^a^ for exons and introns of single-exon and multi-exon circRNAs compared to the values of the corresponding position-adjusted (see ‘Custom reference datasets’ subsection in the Methods) RefSeq exons/introns.

	Genes	Exons						Introns					
		Acceptor^b^		Internal		Donor		Acceptor		Internal		Donor	
HEK 293	RefSeq single-exon	N/A		0.0355		N/A		0.0258		N/A		0.0179	
				0.0225				0.0097				0.0071	
	CircRNAs single-exon	0.0339	**3.2E-1**	N/A		0.0339	**3.2E-1**	0.0109	**2.1E-11**	N/A		0.0087	**5.5E-05**
		0.0224				0.0224		0.0043				0.0047	
	RefSeq multi-exon	0.0380		0.0293		0.0305		0.0295		0.0168		0.0162	
		0.0250		0.0200		0.0200		0.0103		0.0069		0.0068	
	CircRNAs multi-exon	0.0510	**<2.2E-16**	0.0449	**<2.2E-16**	0.0438	**<2.2E-16**	0.0147	**2.1E-3**	0.0125	**1.5E-1**	0.0097	**4.5E-1**
		0.0357		0.0290		0.0309		0.0093		0.0071		0.0065	
HeLa	RefSeq single-exon	N/A		0.1862		N/A		0.1435		N/A		0.0958	
				0.1050				0.0656				0.0496	
	CircRNAs single-exon	0.2289	**1.7E-1**	N/A		0.2289	**1.7E-1**	0.0684	2.6E-5	N/A		0.0556	**4.0E-4**
		0.1198				0.1198		0.0374				0.0340	
	RefSeq multi-exon	0.1741		0.1585		0.1511		0.1338		0.0826		0.0801	
		0.1000		0.0926		0.0900		0.0609		0.0447		0.0437	
	CircRNAs multi-exon	0.2360	**9.6E-13**	0.1781	**1.5E-1**	0.1910	3.5E-6	0.0673	<2.2E-16	0.0526	**<2.2E-16**	0.0533	**<2.2E-16**
		0.1382		0.1009		0.1084		0.0365		0.0252		0.0279	

^a^Average (top) and median (bottom) values. ^b^Bold numbers show P-values of Mann-Whitney U test between the relevant features of circRNAs and the corresponding position-adjusted RefSeq feature.

Measured along the entire body of genes, circRNA-producing genes showed significantly higher elongation speed (*e*.*g*. average Pol II occupancy per nucleotide (Pol II/nt) is lower by ~2.4-fold in HEK 293 and ~1.7-fold in HeLa cells; Supplementary Fig. S9 and Table S5). Part of this difference is due to circRNA-producing genes being longer than average (*c*.*f*. Figs 3e and S4e) and therefore comprising a higher proportion of intronic sequence (*e*.*g*. 96.14% versus 93.55% for HEK 293 cell circRNA genes and the expression-adjusted RefSeq genes). It is known that elongation proceeds more quickly through introns than exons^52,69,72–74^. Calculation of elongation speed separately for introns and exons clearly reproduced this general pattern (*e*.*g*. average Pol II/nt differs between exons and introns by 3.3-fold and 3.0-fold, respectively, for the RefSeq reference gene sets in HEK 293 and HeLa cells; Supplementary Fig. S9 and Table S5). Beyond that, circRNA-producing genes still showed significantly faster elongation along introns (average Pol II/nt ~1.4-fold and ~1.6-fold lower for HEK 293 and HeLa cells, respectively). Exons of circRNA-producing genes showed divergent Pol II speed patterns at the verge of statistical significance: first and last exons tended to show faster elongation, while internal exons were transcribed more slowly than the reference (Supplementary Fig. S9 and Table S5).

**Figure 7:**
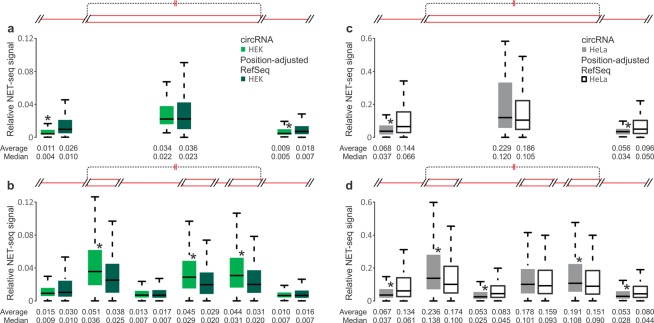
NET-seq signal values (reflecting inverse Pol II speed across mRNA-coding genes) compared between circRNAs of HEK 293 (green; **a**,**b**) or HeLa (grey; **c**,**d**) cells^52^ and the corresponding position-adjusted exons and introns (dark green, white; see ‘Custom reference datasets’ in Methods). (**a**,**c**) Comparisons among the upstream intron, circRNA exon and downstream intron of single-exon circRNAs (left to right; corresponds to the schematic on top). (**b**,**d**) Comparisons among the acceptor intron, acceptor exon, internal intron, internal exon, donor exon and donor intron of multi-exon circRNAs (left to right; corresponds to the schematic on top). Asterisks denote significantly different NET-seq signal between the circRNAs and corresponding exon and intron regions in the reference (P-values < 0.01, Mann-Whitney U test).

Other characteristics of Pol II elongation kinetics still affect these comparisons. For example, it is known that Pol II continues to accelerate along genes^74^. Analyses of the present NET-seq data illustrates this: there is a strong decline in Pol II occupancy from exons 1 to 3 with continuing significant decreases up to at least ordinal position 15 (Supplementary Fig. S10a–c); a similar pattern is seen for introns (Supplementary Fig. S10d–f). This is reflected in a gradual decrease of the NET-seq signal when acceptor and donor exons and introns of multi-exon circRNAs are compared (Supplementary Fig. S11). Thus, we prepared ordinal-position matched reference sets of exons and introns from expression-adjusted RefSeq genes (see ‘Position-adjusted dataset’ in the ‘Custom reference datasets’ subsection in the Methods), one for each specific locus in circRNA-producing genes (Table 2). A clear picture emerges for both single and multi-exon circRNAs: faster Pol II speed through introns is paired with more pronounced slow-down at exons of circRNA-producing genes. This is seen for both, HEK 293 and HeLa cells, with strong statistical significance for many of the pairwise comparisons (Table 2; Fig. 7).

**Figure 8:**
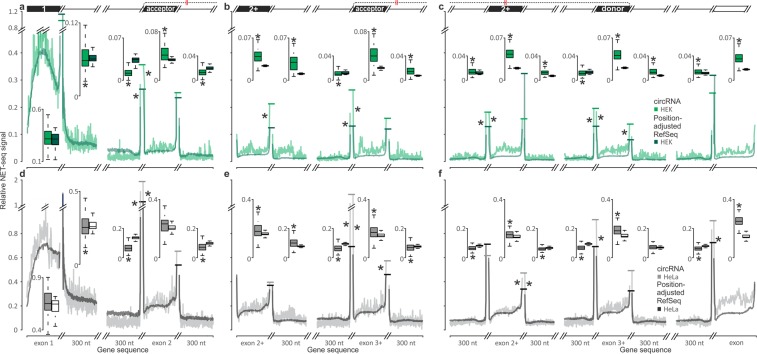
Patterns of NET-seq signal values (reflecting inverse Pol II speed across mRNA-coding genes) compared between circRNAs and the corresponding position-adjusted exons and introns (see ‘Custom reference datasets’ in Methods) in HEK 293 (**a**–**c**) and HeLa (**d**–**f**) cells^52^. NET-seq signals were averaged for different regions of circRNA-producing genes ((**a**–**c**), green line; (**d**–**f**), grey line) and the corresponding position- and expression-adjusted RefSeq average ((**a**–**c**), green line; (**d**–**f**), grey line), as depicted in the schematic on top. NET-seq signal values around acceptor are shown with exon 2 acceptors (**a**,**d**) and with exon 3 or higher acceptors (**b**,**e**). (**c**,**f**) Shows NET-seq signal values around internal circRNA exons and donor exons. To better resolve signals, different discontinuous scaling is used on the X-axis. Coverage of the intronic sequences is represented as an average in each position for a 300 nt region, beginning at the adjacent exon (natural scale). Exonic coverage was first scaled to units of exon length and then averaged (justified scale). Regions focussed on the next upstream or downstream exons are also shown. Signal for regions focussed on circRNA-internal exons were averaged across all instances of their type. Boxplots on the top of the chart represent average NET-seq signal (Pol II occupancy) over the respective regions and have the same scale for each cell type and are aligned by zero for all regions except first introns and exons (position- and expression-adjusted RefSeq values for HeLa are shown as white boxes). Asterisks denote significantly different NET-seq signal between the circRNAs and corresponding regions in the reference (P-values < 0.01, Mann-Whitney U test).

Taking advantage of the nucleotide-level resolution of NET-seq, we also constructed a series of metagene plots to interrogate Pol II occupancy around circRNA-producing gene loci compared to matched regions of expression-adjusted genes (Fig. 8). Specifically, we grouped acceptor exons into those at ordinal position 2 (Fig. 8a,d) and those at position 3 and higher (Fig. 8b,e). We also looked at regions centred at donor exons in position 3 and higher (Fig. 8c,f). Exons were scaled to an arbitrary unit length before plotting of the NET-seq signal, while the adjacent 300 nt of intronic sequences were aggregated directly.

The plots display several previously reported general features of Pol II elongation kinetics: Pol II pausing within the first exon and into the first intron, a faster speed across introns compared to exons and pronounced stall sites at each intron-exon and exon-intron boundary^52,74^. Comparison of circRNA-producing regions to matched counterparts then revealed a more accentuated difference in elongation speed between exons and introns for both HEK 293 and HeLa cells. While first exons were a notable exception, this pattern applied to all internal exons irrespective of their specific role in circRNA production and ordinal position, and arose as a consequence of either a slower passage of Pol II through exons, a faster progress through introns, or both. The characteristic stalling of Pol II at intron/exon and exon/intron boundaries was also more pronounced for circRNA-producing genes. Finally, though discernible on both sides of exons, the speed differential as well as the longer Pol II stalling tended to be more pronounced on their upstream flanks. Our findings are broadly consistent with prior reports that circRNA producing genes are transcribed at a faster-than-average rate^47^. Extending this, we show here that this is primarily driven by a faster intronic elongation rate and in fact coincides with an intricate pattern of slow down at exons.

## Discussion

Interest in the prevalence, function and biogenesis of circular RNAs has risen dramatically in recent years. Here, we have analysed available deep RNA-seq datasets to expand the known circRNA repertoires of human HEK 293 cells^14^ and mouse hearts^57,58^ with over 1,000 new candidates, and to provide a set of over 800 newly identified circRNAs for MEF cells. The status of HEK 293 cells as workhorses for ‘omics’ research then enabled us to assess both, potential functions and aspects of circRNA biogenesis.

With regard to function, we concur with prior studies that evidence for individual circRNAs with an efficient sponge function for a single miRNA or miRNA family is hard to come by and thus likely to be a rare function of circRNAs^2,15^. Nevertheless, we have detected subsets of circRNAs that could collectively have a reasonable miRNA sponge function. One subset could act against the functionally important let-7 family and another against the miR-15/16 family. Given the prominence of these two families, further exploration might be warranted, although experimental follow-up would be difficult as it requires manipulating the levels of multiple circRNAs simultaneously. We further showed that TrIP-seq (and potentially similar) data can be mined for circRNAs that co-sediment with translating ribosomes, allowing us to shortlist ~20% of all detectable circRNAs here as *prima facie* candidates for translation (which we call tc-circRNAs). Additional experimental validation is now required to test whether any of these candidates are truly translated or whether their association with ribosomes, or macromolecular structures of similar sedimentation properties, has other functionality. Translated circRNAs have been shown to contain regions that satisfy functional criteria for internal ribosome entry sites (IRES)^34,35^, and one study provided evidence that the (m^6^A) RNA modification was enriched in circRNAs and served there to recruit the YTHDF3 reader protein, which then attract translation initiation complexes to the circRNA^36^. We focussed here on a comparison of the tc-circRNAs with those circRNAs that lacked evidence of polysome association, to identify features that might promote circRNA translation. However, these efforts failed to detect anything remarkable, which could be because our designated tc-circRNAs included too many false positives and/or false-negatives, or because we did not screen for the appropriate parameter. Although several of the tc-circRNAs overlapped with mapped sites of m^6^A modification, this was not an enriched feature.

We further analysed the physical and functional features of circRNA-producing loci to better understand circRNA biogenesis. In this way, we independently validated, but also integrated and extended, observations made in a number of prior studies (as detailed and referenced above). In broad terms, a multitude of features are associated with the occurrence of circRNAs, either generally at the ‘host gene’ level or more specifically at the level of gene regions directly involved in circRNA formation. Adding to the complexity of data interpretation, these features can often either be interdependent and/or co-occur, as outlined below.

Features that we found to associate with circRNAs at the gene level are (a) a propensity for circRNA-producing genes to be longer and to contain more exons than average, and (b) that they exhibit distinct patterns of Pol II elongation speed. These features might be somewhat interrelated as, for example, Pol II is known to accelerate along the body of genes and to travel faster through genes with long first introns^74^. Conversely, genes that are long overall also tend to have particularly long first introns^68^. Our observation, based on NET-seq data^52^, of an overall faster elongation speed at circRNA-producing genes is consistent with a prior report based on metabolic tagging of nascent RNA^47^. As previously noted, this ties in with observations that co-transcriptional linear splicing is generally favoured by slower Pol II speed^49^ and that manipulating the balance between splicing and elongation rates affects circRNA yields^45,48^. Due to the high resolution of NET-seq we further saw that the faster traverse is limited to introns and contrasted by a slow-down at exons, with enhanced Pol II stalling at intron-exon boundaries. The bigger differential in elongation speed between introns and exons was seen all along circRNA-producing genes and was not limited to the specific circRNA loci. Stalling at boundaries and slower elongation through exons relates to phased nucleosome positioning in DNA^69^ and the incompletely understood ‘exon definition’ mechanism of splicing through cross-exon interactions that later convert to catalytically active cross-intron splicing complexes^71^. Further, a weak Pol II slow-down is characteristic of alternatively-spliced exons; conversely stronger slow-down occurs at constitutively-spliced exons^52^. Our observation that circRNA-producing genes exhibit signs of stronger-than-average exon definition could explain why these genes produce fewer than expected (linear) alternatively-spliced transcript isoforms. Intriguingly, it further suggests an additional cause for the previously observed competition between back-splicing and canonical splicing of pre-mRNA^45^, namely that somehow ‘overactive’ exon definition could hinder linear splicing to favour circRNA formation. Indeed, a similar possibility was raised by data obtained in *Drosophila*, showing that spliceosome depletion shifted the transcription outcome to single exon circRNAs^48^. The authors suggested that under these conditions a direct conversion of cross-exon interactions into catalytically competent back-splicing complexes could occur^2,48^. As our observations hold particularly strongly for multi-exon circRNAs, they indicate that a more complex explanation should be sought and tested experimentally. How exactly exons contribute to an ‘overactive’ exon definition may further differ for single-exon and multi-exon circRNAs, given their marked difference in preferred exon length (see below).

Features that we found to associate with circRNAs directly at the level of gene regions involved in circRNA formation include (a) back-splice acceptor exons are strongly enriched at ordinal position 2 within genes, (b) circRNAs typically span only few exons with two exons being the most prevalent, (c) single-exon circRNAs derive from unusually long exons (while multi-exon circRNAs are generated from exons of more regular length), and (d) flanking introns either side of circRNA loci are unusually long (while circRNA-internal introns are of regular length). Again, these features will be partly interlinked. For example, the first introns of genes are usually much longer than subsequent ones and this effect becomes more pronounced and extends into the second intron for longer genes^68^. Further, first and second introns tend to be spliced more slowly^75^. Concentration of circRNA production around the second linear exon thus may in large part be a consequence of favourable features that are prevalent in this gene region, *e*.*g*. long flanking introns that are spliced less efficiently^45^ or allow Pol II to accelerate towards the end of the long intron and create larger speed difference with the subsequent exon transcription^74,76^. Still, ordinal intron/exon position by itself is neither sufficient nor required to lead to detectable circRNA levels as they can arise from essentially any position within genes. Furthermore, we have shown here that circRNA-flanking introns are not just longer than the general average, but they specifically exceed the intron length that is typical at each given ordinal position within genes. This is likely related to the tendency for transcription and splicing to be co-optimised^77^; thus to lead to circRNA formation, any perturbations of these processes will need to be context-aware.

Evidence exists in favour of both, co- and post-transcriptional formation of circRNAs by back-splicing e.g.^37,45^. Some of the features outlined above would be conducive to back-splicing in either scenario. For example, short exon span and long flanking introns could simply favour back-splicing as they increase the probability of back-spliced acceptor and donor to interact with each other before the canonical linear splice-sites could interact due to the longer distances, in the context of a completed, or near complete, linear pre-mRNA (Supplementary Fig. S12a). In favour of a co-transcriptional mode of circRNA formation is the fact that essentially all features described here can be rationalised in such a model. In different ways, each feature can perturb the balance between transcription and linear splicing to create important preconditions for back-splicing on nascent transcripts (Supplementary Fig. S12b). At the upstream flank of circRNA loci, a combination of extended intron length and an accentuated ramp in Pol II speed at the intron-exon boundary as well as the greater persistence of cross-exon interactions create a delay in linear splicing. In this case, the 5′-flanking intron donor site begins to be processed while Pol II is still *en route* towards the respective acceptor, resulting in the completed assembly of the activated complex B (B*). However, upon transition into complex C there is inefficient resolution of the complex C into a cut-off lariat and linearly spliced exons, eventually resulting in a functional loss of the active 3′ end of the donor and perhaps even some irreversible abortion of linear splicing^78^. Either way, the resultant complex C is then still available to react with the downstream splice-donor when the latter emerges from Pol II; the typically limited exon span of circRNAs will ensure that the time for that is kept short. The longer intron at the downstream flank once again kinetically assist circularisation, by providing additional time prior to the appearance of the ‘canonical’ downstream acceptor exon that would result in linear splicing. Combined with the known means of creating intron-looping interactions (*e*.*g*. through RCMs or RBPs), the scenarios described above could then generate appreciable quantities of circRNA.

The study of circRNA biogenesis and function continues to surprise and delight, and increasing evidence of their involvement in different biological processes provides a broader justification for their continued exploration^23^. The evidence provided here strengthens the case to test for meaningful translation of circRNAs and for a link between Pol II transcription kinetics and circRNA formation. The latter emphasises the more general need to better understand how coupling between elongation and pre-mRNA processing determines splicing outcomes and how this is driven by (local) chromatin architecture.

## Methods

### RNA sequencing data

Ribosome-depleted RNA-seq data for mouse embryonic fibroblasts (E12.5 embryos) (SRA accession numbers SRR2038028, SRR2038029, SRR2038030, SRR2038031)^51^, mouse adult heart (SRR2038032, SRR2038033)^51^, HEK 293 cells (SRA SRR2044110 and SRR2044119)^50^ and polysome fractions of HEK 293 cells (SRA SRR2044111-SRR2044119 and SRR2044120-SRR2044127)^50^ were downloaded from the National Center for Biotechnology Information (NCBI) Short Read Archive (SRA), https://www.ncbi.nlm.nih.gov/sra. SRA files were converted to fastq files using ‘fastq-dump’ program in sratoolkit (version 2.4.4).

### Reference and annotation files

The genome reference files and the Bowtie2 index files (*Mus musculus*; University of California, Santa Cruz (UCSC) 10 mm and *Homo sapiens*; UCSC 19 hg) were downloaded from http://bowtie-bio.sourceforge.net/bowtie2/index.shtml. Annotation files for genome mapping (gencode.vM8.annotation.gtf (10 mm) and gencode.v19.annotation.gtf (19 hg)) were downloaded from GENCODE (part of the Encyclopedia Of DNA Elements (ENCODE) project) depository (https://www.gencodegenes.org/). RefSeq genes and their exon positions in each genome (10 mm and 19 hg) were extracted from RefFlat files downloaded from UCSC genome browser (http://hgdownload.soe.ucsc.edu/downloads.html).

### Custom reference datasets

#### Non-circRNA-producing gene set

Is a set of mRNA-producing RefSeq genes resulting in ≥1 FPKM of the RNA-seq data in the same cell/tissue as the detected back-spliced junctions, but excluding genes producing the back-spliced junctions.

#### Exon-count-adjusted gene set

Is a set of RefSeq genes which were randomly selected to match the numbers of exons in the genes producing back-spliced junctions.

#### Gene-length-adjusted gene set

Is a set of RefSeq genes which were randomly selected to match the lengths of genes producing the back-spliced junctions.

#### Cell-specific (HEK-, MEF- and MH-specific) gene set

Is a set of RefSeq genes which sequences were detected with at least 1 FPKM in the corresponding RNA-seq data.

#### Expression-adjusted gene set

Is a set of cell-specific genes that do not produce back-spliced junctions and were randomly selected to match the abundance of RNA synthetized from the genes that produce back-spliced junctions.

#### Position-adjusted set

Is a set of RefSeq exons/introns assembled from the corresponding expression-adjusted gene set where the exons/introns were randomly selected to match the positional distribution of the corresponding feature of genes relative to the location of the back-spliced junction (and the putative structures of circRNA-producing genes). *E*.*g*., in Fig. 7, the position-adjusted set included cell-specific gene set which was randomly selected to result in a similar average ordinary position frequencies of the corresponding features (introns and exons) of single-exon and multi-exon mRNAs; in Fig. 8b, for circRNAs with acceptors located at positions 3 and more of the genes, the ratio of the exon position frequencies in the position-adjusted set is matching at exon3:0.26, exon4:0.16, exon5:0.12, *etc*.).

### Identification of circRNAs in paired-end sequencing reads

The overall strategy employed was similar to the previously used circRNA detection routine based on the unique mapping of the back-spliced junctions (Fig. 1a)^9^. To identify putative circRNA reads, first all reads that map to the canonically spliced exons of the reference genome were discarded. Next, all reads with non-canonical reference exon order were detected. These were then searched for containing at least one read of the pair to intersect and reliably map across a back-spliced exonic junction. The second read of the pair was then confirmed to be located within the boundaries of the circRNA as defined by the back-spliced junction read.

#### Read mapping to reference genome

Mouse (mouse embryonic fibroblast and heart) and human (HEK 293 cells) reads were mapped to the reference genomes 10 mm and 19 hg, correspondingly, using Bowtie2 (version 2.2.6)^79^ and Tophat2 (version 2.1.0) combination^80^ and pre-built annotation files (GENCODE files mentioned above in the ‘Reference and annotation files’ subsection). Default performance parameters were used for both programs except ‘-b2-sensitive’. For example: tophat-b2-sensitive -p xx [# of processors] -G [annotation.gtf] -o [output dir] read1.fastq read2.fastq.

#### Detection of non-canonical splice junctions

To detect reads that contain unconventional splice-sites including back-spliced exon-exon junctions, ‘fastq’ files for the unmapped paired-end reads were assembled from Tophat2 ‘unmapped reads’ and mapped again to the same reference genome using Tophat2 with ‘fusion-search’ parameter^81^. For example: tophat-fusion-search-b2-sensitive -p xx [# of processors] -G [annotation.gtf] -o [output dir] unmapped-read1.fastq unmapped-read2.fastq.

#### Detection of back-spliced exon-exon junctions

Reads containing back-spliced junctions at least in one read of the pair were next identified in the mapped reads from Tophat2 fusion-search routine. Separately, loci of exon start and end positions, exon numbers, mRNA identifiers (IDs) and gene names were extracted from RefFlat files and converted to ‘bed’ format files. The reads containing back-spliced junctions were mapped to the RefSeq annotation files using ‘intersectBed’ program in BedTools (version 2.25.0)^82^.

#### Verification of circRNAs and normalisation of the read counts

The resultant paired-end reads containing back-spliced junctions were selected if the back-spliced junction read overlapped with the start/end positions of the RefSeq exons. These hits were counted as predicted circRNA only if the other read of the pair was verified to be inside of the anticipated RNA circle. The source codes for the ‘back-spliced junction detection’ are available from GitHub (https://github.com/raganc/exonicCircularRNA).

Abundance levels of circRNAs were normalised by dividing the number of the back-spliced junctions (measured as JPM) by the number of the mapped reads (measured as RPM), individually for each sample. We used a threshold for an average sample abundance as ≥0.1 JPM for each cell or tissue type. In HEK 293 cells, 18 circRNAs were present with the average of <0.1 JPM in total cytoplasmic RNA sequencing data, but were ≥0.1 JPM abundant across the eight polysome fractions (see Fig. 2a and ‘Detection of circRNAs in ribosome sedimentation profiles’ subsection below). Therefore, we included these circRNAs in the HEK 293 detection lists.

### Back-spliced acceptor and donor exon positions in circRNA genes

Favourable positions of acceptors and donors were computed by dividing the number of acceptors and donors in each exon position by the numbers of total circRNA exons (for the non-adjusted values), or dividing the number of acceptors and donors in each exon position by the number of RefSeq exons in the same exon position, then the resultant ratio of each exon position was divided again by the sum of occurrence in each position (normalized by RefSeq exon numbers). Normalization by circRNA exon numbers was calculated using the occurrence of circRNA exons at specific positions instead of all RefSeq exons as the divider.

### Lengths of introns in circRNA genes

Lengths of RefSeq introns were computed from the exon end and start positions in RefFlat files. The average lengths of upstream acceptor, downstream donor, and internal introns of circRNAs genes in different positions (*e*.*g*. intron 1, intron 2, … intron *n*) were compared to the average lengths of the corresponding intron positions of all annotated RefSeq mRNAs. Since in most cases it was not possible to detect from which mRNA isoform circRNAs were produced, we included all isoforms fully covering circRNA extent for the circRNA measurements and all annotated RefSeq isoforms for the total RefSeq mRNAs.

### Transcript variance (isoforms) and abundance of linear RNA transcripts

Abundance levels and transcript variances of cognate linear mRNAs were identified from the outputs of Tophat2 mapped reads (first step of read mapping in Fig. 1a; see ‘Read mapping to reference genome’) using cuffquant and cuffnorm programs in Cufflinks package (version 2.2.1)^83,84^ and a gene annotation and a repeat sequence masking file (see below). For example: cuffquant -o [output directory] -M 10 mm-msk.gtf (or 19 hg-rmsk.gtf) annotation.gtf[same as tophat] accepted_hits.bam[tophat output], and cuffnorm -o [output directory] annotation.gtf[same as tophat] xxx.cxb1, xxx.cxb2, … [output files] (from each sample) from cuffquant.

The masking file (masked loci of rRNA, tRNA and mitochondrial chromosome (chrM) transcripts) was obtained from USCS genome Table Browser (https://genome.ucsc.edu/cgi-bin/hgTables), as per following examples. For rRNA and tRNA, assembly: 10 mm, group: all tables, output format: gtf, table: rmsk, filter: create – repClass: rRNA, free-form query: rRNA or tRNA. For chrM, the following was substituted. Select table: knownGene, filter: create – chrom: chrM. The resultant files then were combined into one ‘gtf’ file.

We used a threshold of an average abundance ≥1 fragments per 1,000 bases of exon per million reads mapped (FPKM) in each sample, which is typically used in total RNA-seq data analyses^83^. To adjust transcript length and number of exons of the circRNA non-producing genes to match those of the circRNA-producing genes, we randomly selected genes with a similar numbers of exons and lengths (see ‘Custom reference datasets’ subsection).

### Analysis of the read distribution for Pol II-synthesised nascent RNA

NET-seq and the corresponding total RNA-seq data (accession numbers GSM1505440, GSM150544144 in ‘bedgraph’ format and SRR1928210) for HEK 293 cells and (accession numbers GSM1505438, GSM1505439 in ‘bedgraph’ format and SRR1575938) for HeLa S3 cells were downloaded from NCBI website (http://www.ncbi.nlm.nih.gov/geo/)^52^. The data were analysed using human genome 19 hg as reference.

NET-seq datasets derived from two biological replicates were combined by taking the average of the read counts. Because the number of circRNAs was small compared to RefSeq exons, to avoid skewing of the results by outliers, we limited the maximum nascent RNA read counts to 10 in a single locus. ~0.03% of exon/intron regions show more than 10 read counts where the average count is ~80 (maximal observed count is ~25,000), the nascent RNA read counts in these loci were adjusted to 10.

To obtain relative coverage of RefSeq exons and introns, we first mapped reads from the RNA-seq data using Star aligner version 2.5.2b^85^ with the parameters set described in the original NET-seq study^52^. After removal of reads mapped to rRNAs, tRNAs, mitochondrial genome and repeat sequences, ~240 million reads (of ~470 million of initial reads) in HEK 293 and ~36 million reads (of ~57 million of initial reads) in HeLa S3 datasets were uniquely mapped to the reference genome. Expressed RefSeq genes were detected with ≥1 RPM cut-off. We then mapped the reads to RefSeq exons using BedTools^82^, identified ~103,000 unique exon loci in HEK 293 and ~95,000 unique exon loci in HeLa S3 cells, and also inferred intronic data from these exon coordinates.

To count Pol II coverage in circRNA-producing genes for HEK 293 cells, we used set of circRNAs detected in this study (Supplementary Table S1). For HeLa S3, we used 854 unique circRNAs predicted in HeLa cells previously^86^. Nascent RNA read hits were next counted if they overlapped with each of the circRNA exons of interest and 300 nt of the upstream and downstream (adjacent) introns from the start and end of the exons. To obtain the nascent RNA read density scaled to exon length, the read coverage on each exon was justified by the length of the exon.

### Detection of circRNAs in ribosome sedimentation profiles

We used sedimentation-resolved RNA-seq data which contained rRNA-depleted RNA sequences of each of eight ribosomal fractions (monosomes, disomes, *etc*. up until fraction containing octasomes and all and faster sedimenting polysomes), and the corresponding total cytoplasmic RNA control data^50^. CircRNAs and linear RNAs in HEK 293 were identified as mentioned above. Relative abundance (proportion) of the ribosome and polysome non-associated circRNAs and their cognate linear counterparts were calculated as follows. Ribosome non associated (Free*) = (Total cytoplasmic × 9) − (monosomes + … + octa(+)somes) (JPM).

Using custom code available at GitHub (https://github.com/raganc/exonicCircularRNA), we identified the respective start codons and start codon nucleotide context in the spliced exons spanning circRNAs, including over back-spliced junction. For ORF statistics, all three stop codons (UAG, UAA, UGA) were identified in-frame with the detected start sites using three rounds of the entire sequence of the spliced exons after the back-spliced junction. The presence of an infinite loop ORF within circRNA was inferred if none of the stop codons appeared in-frame.

Positions of m^6^A methylation sites were retrieved from the available data on single-nucleotide resolution positions of m^6^A in poly(A)^+^ RNA of HEK 293 cells^87^. We combined 9,536 putative m^6^A sites identified by cross-linking induced mutation CLIP and 6,543 sites identified by cross-linking induced truncation CLIP^87^. The m^6^A sites overlapping with circularized and non-circularized exons were identified with ‘intersectBed’ program of BedTools package ran with default parameters^82^.

### Detection of RBP binding sites

Positions of known RBP binding sites mapped in HEK 293 cells were downloaded from CLIPdb database (http://lulab.life.tsinghua.edu.cn/clipdb/)^60^ and starBase v2.0 database (http://starbase.sysu.edu.cn/index.php)^61^. The positions included binding sequences of four Ago protein family members and 39 other RBPs, identified in various CLIP-seq experiments (Supplementary Fig. S2a). The sequences of the corresponding binding sites in 19 hg genome loci in the form of ‘bed’ file were mapped to RefSeq exons using ‘intersectBed’ program of the BedTools package^82^, specifying fraction as 75% (*i*.*e*. ≥75% of the binding site sequences have to be overlapped with exons). The number of binding sites identified for each RBP was compared between circularized exons and non-circularized exons using two-sample proportion test (‘prop.test’) with P-value < 0.05 in the R software package version 3.1.3 (https://www.r-project.org/) (Supplementary Fig. S2b).

### Detection of miRNA binding sites

Sequences of AGO proteins (AGO1-4) binding sites in circRNA exons were obtained as mentioned above in the ‘Detection of RBP binding sites’ subsection. 2,588 human mature miRNA sequences (‘mature.fa’) were retrieved from miRBase database release version 21 (http://www.mirbase.org/ftp.shtml).

The miRNA targets sites in circularized exons were predicted using the oligoarrayaux-3.8 program^88^ downloaded from The DINAMelt Web Server, The RNA Institute, College of Arts and Sciences, State University of New York at Albany (NY) (http://unafold.rna.albany.edu/?q=DINAMelt/OligoArrayAux), using parameters: hybrid-min–suffix = DAT–mfold = 50,5,100–maxloop = 8. All predicted mature miRNA-exon binding sites were filtered to have Watson-Crick base pair seed match at positions 2–7 nt from the 5′ ends of miRNAs. These predicted target sites were then searched for overlap with the AGO binding sites plus 10 nt up and downstream from them using ‘intersectBed’ program in BedTools package^82^, specifying fraction as 50% (~20 nt in length).

## Supplementary information

Supplementary Information

Supplementary Table S1

Supplementary Table S2

Supplementary Table S3

## Data Availability

The source RNA-seq data used are publicly available under the respective accession numbers, as indicated. The code used to identify circRNAs and locate and characterise start sites and ORFs is available at GitHub (https://github.com/raganc/exonicCircularRNA).
