# Supplementary material for: Insights into the biogenesis and potential functions of exonic circular RNA
Source: Sci Rep. 2019 Feb 14;9:2048. doi: 10.1038/s41598-018-37037-0 (PMC6376117; doi:10.1038/s41598-018-37037-0)
Supplement: Supplementary file 1 — Supplementary Information [file 41598_2018_37037_MOESM1_ESM.pdf]

SUPPLEMENTARY INFORMATION
for the manuscript

Insights into the biogenesis and potential functions of exonic circular RNA

Chikako Ragan[1], Gregory J. Goodall[2,3,4], Nikolay E. Shirokikh[1*], Thomas Preiss[1,5*]

1. EMBL–Australia Collaborating Group, Department of Genome Sciences, The John Curtin School of Medical Research, The Australian National University, Canberra, ACT 2601, Australia
2. Centre for Cancer Biology, University of South Australia and SA Pathology, Adelaide, SA, 5000, Australia
3. Discipline of Medicine, The University of Adelaide, Adelaide, SA 5005, Australia
4. School of Molecular and Biomedical Science, The University of Adelaide, Adelaide, SA 5005, Australia
5. Victor Chang Cardiac Research Institute, Darlinghurst, NSW 2010, Australia

* corresponding author Thomas Preiss Thomas.Preiss@anu.edu.au

* corresponding author Nikolay E. Shirokikh nikolay.shirokikh@anu.edu.au

Results

Computational prediction of human and mouse circRNAs

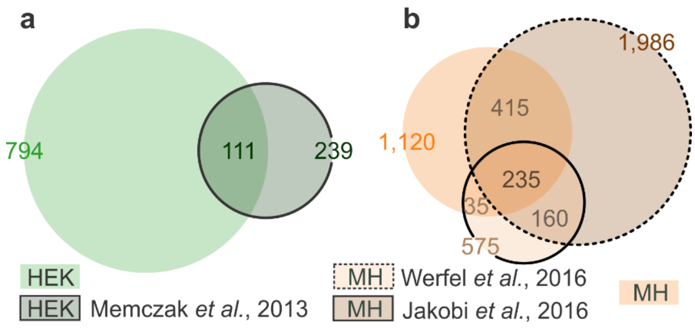

Supplementary Figure S1. Comparison with other circRNA predictions. Sets of circRNAs from mouse heart (a) and HEK 293 cells (b) reported here based on data from ref. 51 were intersected with sets from previously published works[14,58,59] using the equivalent source material.

Assessing circRNA potential as miRNA sponges

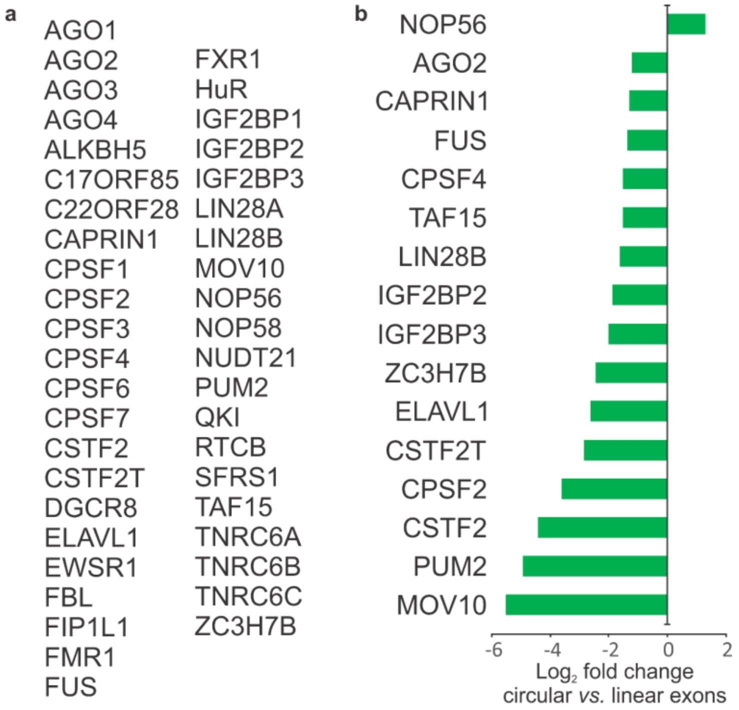

Supplementary Figure S2. Analysis of the occurrence of RNA-binding proteins (RBPs) sites in the spliced exonic circRNAs detected in HEK 293 cells. (a) List of RBPs with extensive CLIP-seq coverage[61,62]. (b) Relative frequency of the RBP sites demonstrating significant difference in either direction of abundance between circRNA exons compared to the linear mRNA exons (Fisher's exact test with false discovery rate <0.05 cut-off).

Identifying circRNAs with potential for translation

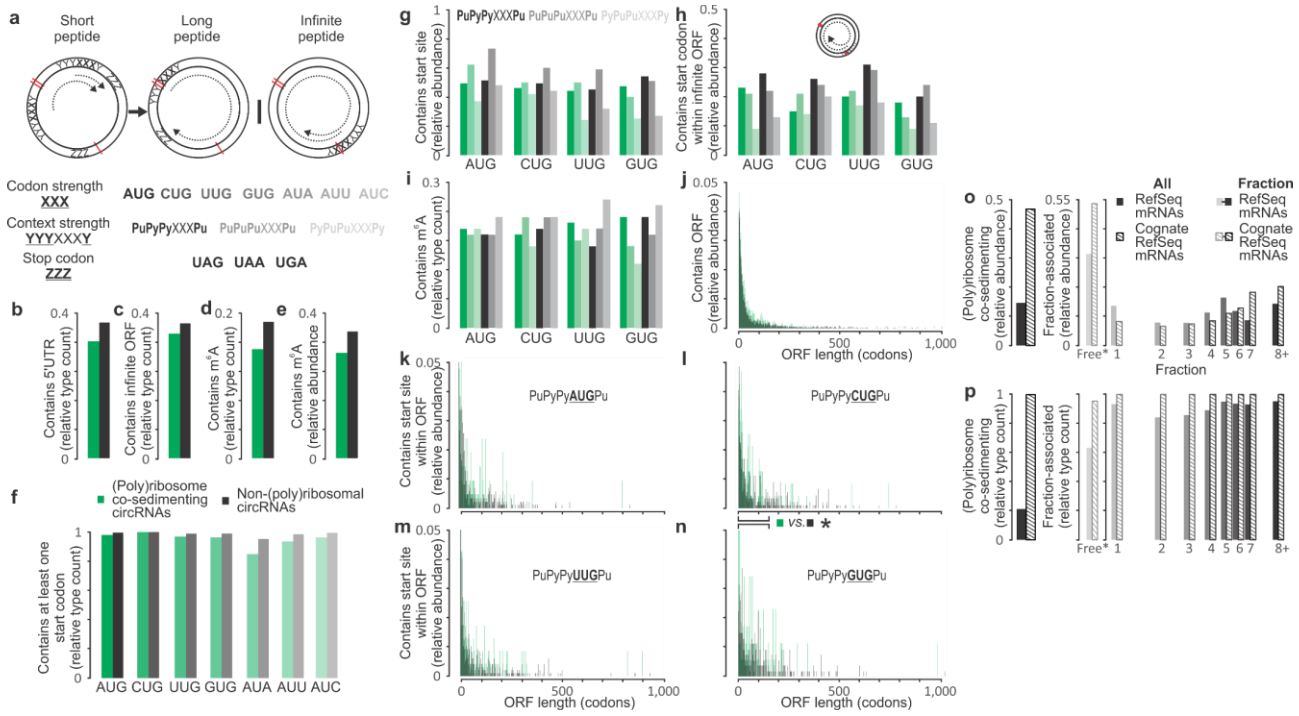

Supplementary Figure S3. Features of human translation candidate (tc-)circRNAs. See main text Fig. 2 and the accompanying text for the detection of HEK 293 cell tc-circRNAs. (a) Schematic of different analysed circRNA features that can affect their translation potential, including ORF length (gradually increasing and infinite) and count, strength of initiation codon and start codon nucleotide (Kozak's) context[90-92]. All three stop codons in the same reading frame as the start codon were used to locate ORFs in circRNA. (b) Prevalence of 5' UTR sequences in (poly)ribosome co-sedimenting and non-associated circRNAs measured by the presence of circRNAs (counts of unique back-spliced junctions). (c) Same as (b), but for at least one infinite ORF. (d) Same as (b), but for at least one m^{6}A site. (e) Same as (d), but measured by relative frequency of m^{6}A sites across back-spliced junctions. (f) Occurrence of at least one of the start codons of different strengths in (poly)ribosome co-sedimenting and non-associated circRNAs by the presence of circRNAs (counts of unique back-spliced junctions). (g) Frequency of the start codons combined with the three types of nucleotide context strength in (poly)ribosome co-sedimenting and non-associated circRNAs measured across back-spliced junctions. (h) Same as (g), but for infinite loop ORFs only. (i) Same as (g), but for occurrence of at least one m^{6}A site and measured by the presence of circRNAs (counts of unique back-spliced junctions). (j-n) Occurrence of ORFs (j) and indicated start sites (k-n) (Y-axis) in ORFs of different lengths (X-axis) in (poly)ribosome co-sedimenting and non-associated circRNAs. (j) For all ORFs; (k-n) for ORFs beginning with the indicated start site. (l,n) Occurrence of CUG and GUG codons in strong nucleotide context across ORFs of different lengths are significantly different (P-value <0.01, Mann-Whitney U test) between (poly)ribosome co-sedimenting and non-associated circRNAs. (o,p) Same as Fig. 2b,c, but depicting counts for RefSeq mRNAs instead of circRNA-attributed reads.

CircRNA-producing genes have a splice-isoform-generating capacity comparable to genes of similar structure

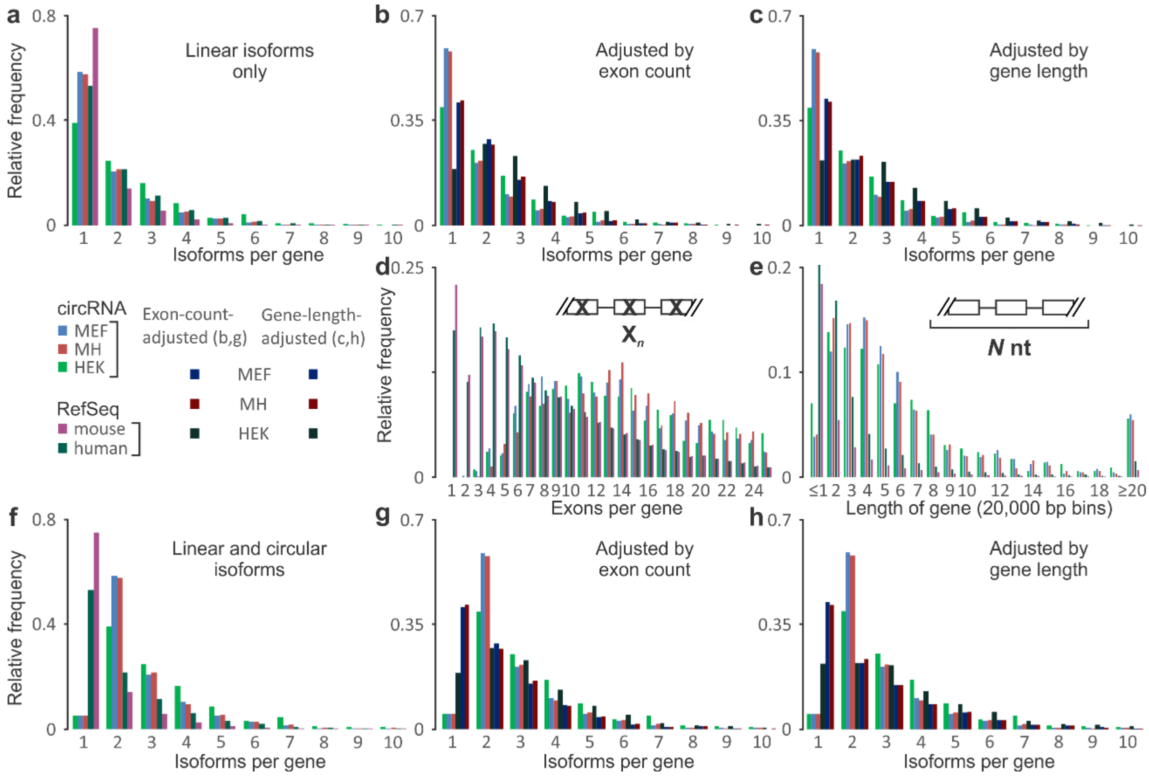

Supplementary Figure S4. Linear isoform diversity of human and mouse circRNA-producing genes. (a) mRNA isoform frequency of circRNA-producing genes compared to RefSeq genes. (b) Same as (a), but compared to exon-count-adjusted genes. (c) Same as (b), but compared to gene-length-adjusted genes. (d) Exon counts in circRNA-producing genes compared to RefSeq genes. (e) Same as (d), but for gene lengths (binned in 20,000 bp steps). (f-h) Same as (a-c), but circRNAs are also counted as transcript isoforms produced from each gene. Designations and abbreviations as in Fig. 1 and Fig. 3. The distribution of isoforms (a, f, g, h), number of exons (d) and length of genes (e) are significantly different between circRNA-producing genes and reference genes (P-value <0.01, Mann-Whitney U test).

CircRNAs typically have a short exon span and include exons that emerge early during transcription

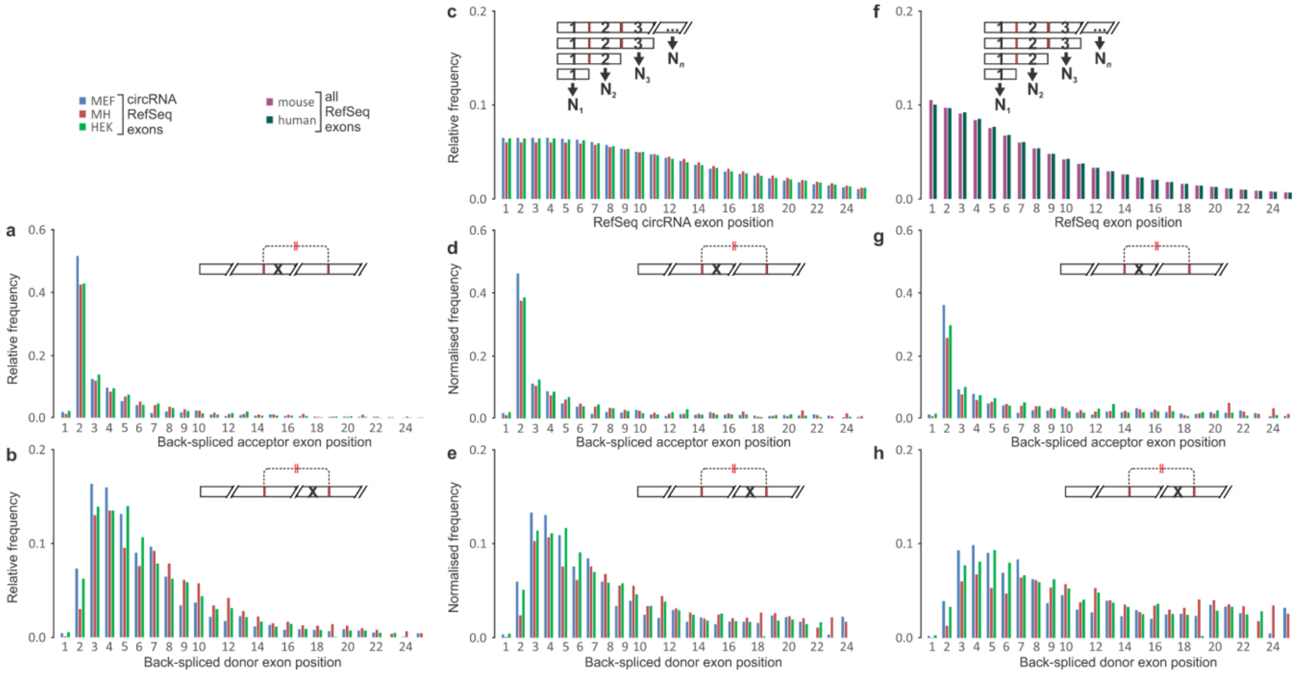

Supplementary Figure S5. Exon position bias at human and mouse circRNA-producing gene loci. (a) Relative frequency of back-spliced acceptor exon positions in circRNA-producing genes. Acceptor exons were over-represented in the second position of circRNA-producing genes compared to other exon positions (P-value <0.01, Mann-Whitney U test). (b) Same as (a), but for back-spliced donor exon positions. (c) Relative frequencies of exons by ordinal position along circRNA-producing genes. (d) Same as (a), but after normalisation to exon frequency as shown in (c). Acceptor exons were over-represented in the second position of circRNA-producing genes compared to other exon positions even after normalisation by (c) (P-value <0.01, Mann-Whitney U test). (e) Same as (b), but after normalisation to exon frequency as shown in (c). (f) Same as (c), but for all RefSeq annotated genes. (g,h) Same as (d,e), but after normalisation to exon frequency as shown in (f). (g) Acceptor exons were over-represented in the second position of circRNA-producing genes compared to other exon positions even after normalisation by (f) (P-value <0.01, Mann-Whitney U test). Note: where applicable, each RefSeq annotated mRNA was considered as a potential cognate linear isoform for a given circRNA. This 'inclusive' approach was used to avoid bias; however, an additional effect is the low frequency of erroneous acceptor and donor assignment to exon position 1. Designations and abbreviations as in Fig. 1 and Fig. 4 with the following additions: mouse RefSeq, human RefSeq.

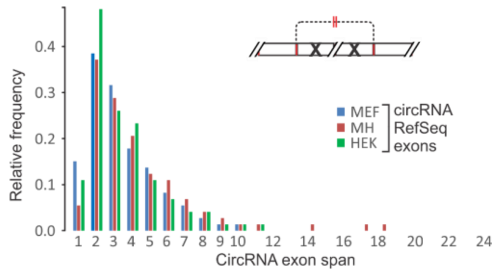

Supplementary Figure S6. Human and mouse circRNA exon span. Note: where applicable, each RefSeq annotated mRNA was considered as a potential cognate linear isoform for a given circRNA. Designations and abbreviations as in Fig. 1 and Fig. 4.

CircRNA-generating exons tend to be of untypical lengths

Supplementary Table S4. Lengths values[a] for exons and introns of circRNAs compared to the values of the cell-specific gene set. [a]Average (top) and median (bottom) length values in nucleotides. [b]Cell-specific gene set included RefSeq genes which sequences were detected in the respective RNA-seq data (see 'Custom reference datasets' subsection of the Methods for details). [c]Faded-out numbers show P-values of Mann-Whitney U test between the distributions of relevant feature of circRNAs and the internal exons (the first and last exons were removed) and introns of expressed RefSeq genes.

	Genes[b]	Exons						Introns					
		Acceptor[c]		Internal		Donor		Acceptor		Internal		Donor	
MEF	RefSeq	N/A		148.6<br>121		N/A		N/A		4,879.8<br>1,376		N/A	
MEF	Single-exon circRNAs	539.7<br>248	< 2.2E-16<br>< 2.2E-16	N/A		539.7<br>248	< 2.2E-16<br>< 2.2E-16	26,683.6<br>15,198	< 2.2E-16<br>< 2.2E-16	N/A		23,109.4<br>12,182	< 2.2E-16<br>< 2.2E-16
MEF	Multi-exon circRNAs	165.0<br>119	9.0E-1<br>1.7E-1	142.4<br>115	1.3E-4<br>1.6E-6	196.6<br>126	9.0E-5<br>3.0E-4	23,373.2<br>17,224	< 2.2E-16<br>< 2.2E-16	4,873.5<br>2,439	< 2.2E-16<br>< 2.2E-16	21,394.5<br>11,555	< 2.2E-16<br>< 2.2E-16
MH	RefSeq	N/A		149.3<br>121		N/A		N/A		4,695.4<br>1,359		N/A	
MH	Single-exon circRNAs	404.0<br>169	2.7E-8<br>2.9E-8	N/A		404.0<br>169	2.7E-8<br>2.9E-8	20,144.1<br>5,804	1.1E-10<br>1.1E-9	N/A		16,897.9<br>5,315	4.5E-13<br>3.4E-10
MH	Multi-exon circRNAs	171.1<br>119	8.0E-1<br>4.3E-1	140.9<br>117	3.1E-5<br>1.4E-6	170.9<br>120	7.5E-1<br>5.9E-1	19,162.1<br>11,579	< 2.2E-16<br>< 2.2E-16	3,927.6<br>1,942	< 2.2E-16<br>< 2.2E-16	16,371.0<br>9,305	< 2.2E-16<br>< 2.2E-16
HEK 293	RefSeq	N/A		148.3<br>121		N/A		N/A		5,825.1<br>1,541		N/A	
HEK 293	Single-exon circRNAs	708.9<br>253	< 2.2E-16<br>< 2.2E-16	N/A		708.9<br>253	< 2.2E-16<br>< 2.2E-16	19,985.0<br>8,413	< 2.2E-16<br>< 2.2E-16	N/A		17,530.5<br>5,898	< 2.2E-16<br>1.1E-14
HEK 293	Multi-exon circRNAs	164.7<br>124	1.1E-1<br>4.4E-2	132.5<br>115	4.8E-7<br>5.8E-9	159.7<br>117	7.6E-2<br>5.8E-3	20,627.6<br>11,761	< 2.2E-16<br>< 2.2E-16	4,890.8<br>2,394	< 2.2E-16<br>< 2.2E-16	20,433.4<br>11,216	< 2.2E-16<br>< 2.2E-16

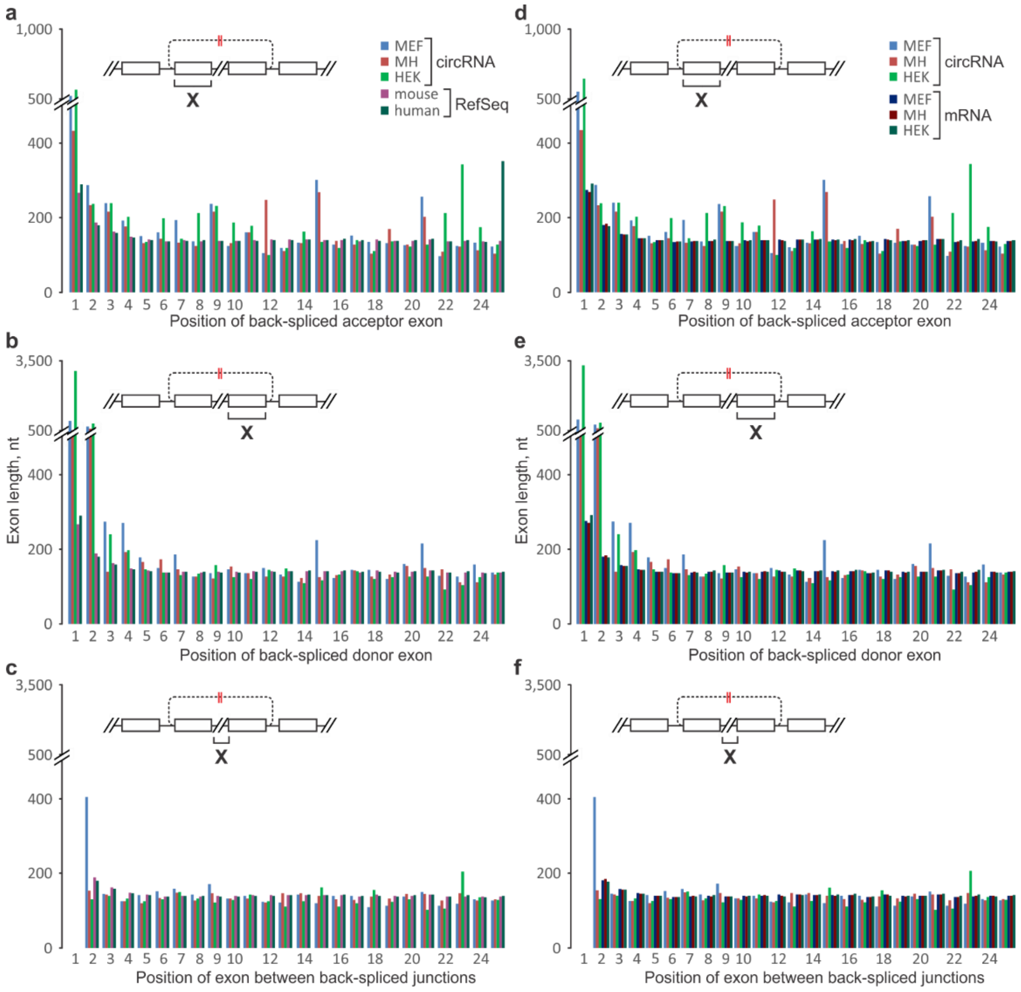

Supplementary Figure S7. Exon lengths at mouse MEF, MH and human HEK 293 cell circRNA loci. Exons from circRNA-producing genes are compared to RefSeq (a-c) or cell-specific RefSeq (d-f) averages in the corresponding linear positions. (a) Average back-spliced acceptor exon lengths. (b) Same as (a), but for the back-spliced donor exons. (c) Same as (a), but for circRNA-internal exons. (d-f) Same as (a-c), but the controls include only mRNAs detected in the respective cell-specific RefSeq datasets (see 'Custom reference datasets' subsection in the Methods). Designations and abbreviations are as in Fig. 1 and Fig. 5 with the following additions: mouse RefSeq, human RefSeq. Note: introns located between exons 1 and 2 of each RefSeq-annotated transcript were denoted as intron 1. Where applicable, all RefSeq annotated mRNAs (linear isoforms) overlapped with a circRNA were considered as potential cognate linear isoforms for a given circRNA.

CircRNA back-splicing acceptor and donor sites are typically flanked by long introns

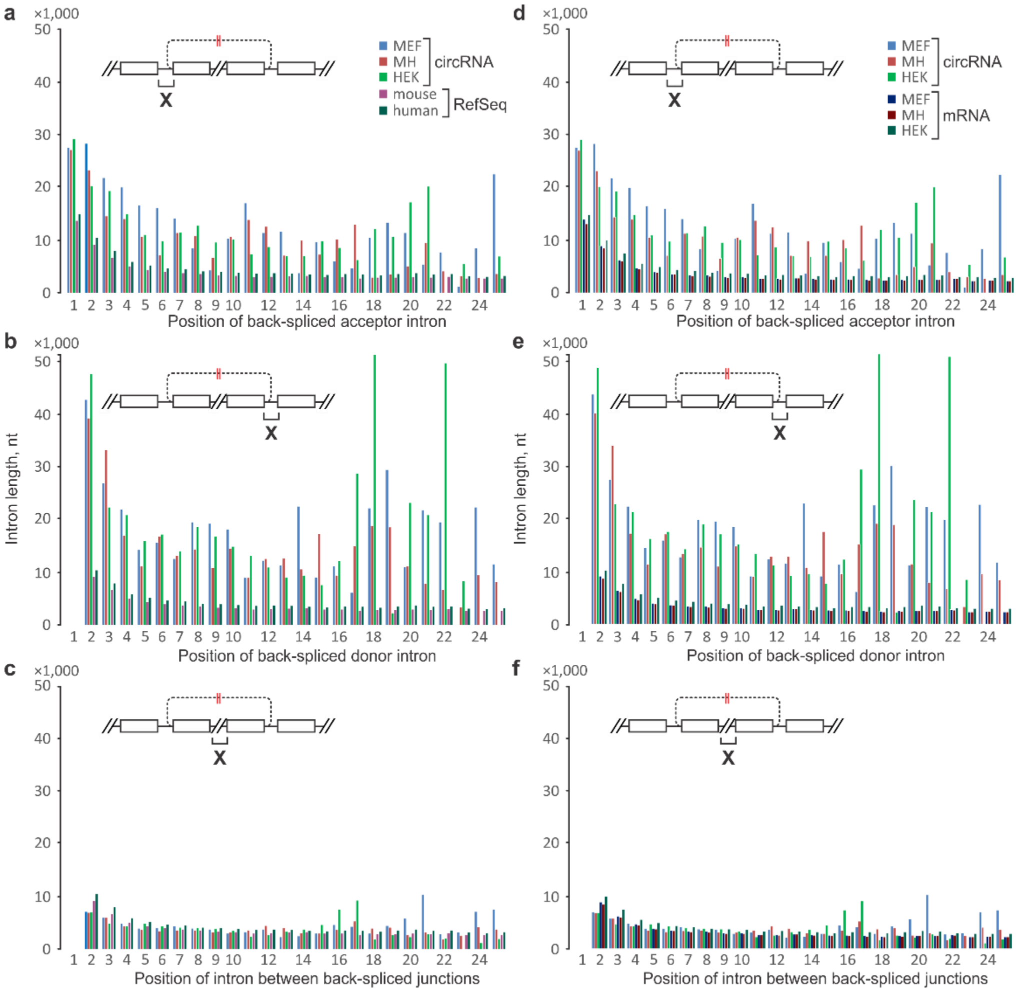

Supplementary Figure S8. Intron lengths at human and mouse circRNA-producing gene loci. Introns from circRNA-producing genes are compared to RefSeq (a-c) or cell-specific RefSeq (d-f) averages in the corresponding linear positions. (a) Average intron lengths at the upstream flank of back-spliced acceptor exons. (b) Same as (a), but for the introns at the downstream flank of back-spliced donor exons. (c) Same as (a), but for circRNA-internal introns. (d-f) Same as (a-c), but the controls include only mRNAs detected in the respective cell-specific RefSeq datasets (see 'Custom reference datasets' subsection in the Methods). Designations and abbreviations are as in Fig. 1 and Fig. 6 with the following additions: mouse RefSeq, human RefSeq. (d,e) Acceptor and donor introns of circRNA-producing genes are much longer than RefSeq genes in same ordinal positions and overall lengths of acceptor and donor introns were significantly longer than introns of RefSeq genes (measured by P-value <0.01, Mann-Whitney U test). Note: introns located between exons 1 and 2 of each RefSeq-annotated transcript were denoted as intron 1. Where applicable, all RefSeq annotated mRNAs (linear isoforms) overlapped with a circRNA were considered as potential cognate linear isoforms for a given circRNA.

Accentuated differences in transcription speed between introns and exons are features of circRNA-producing genes

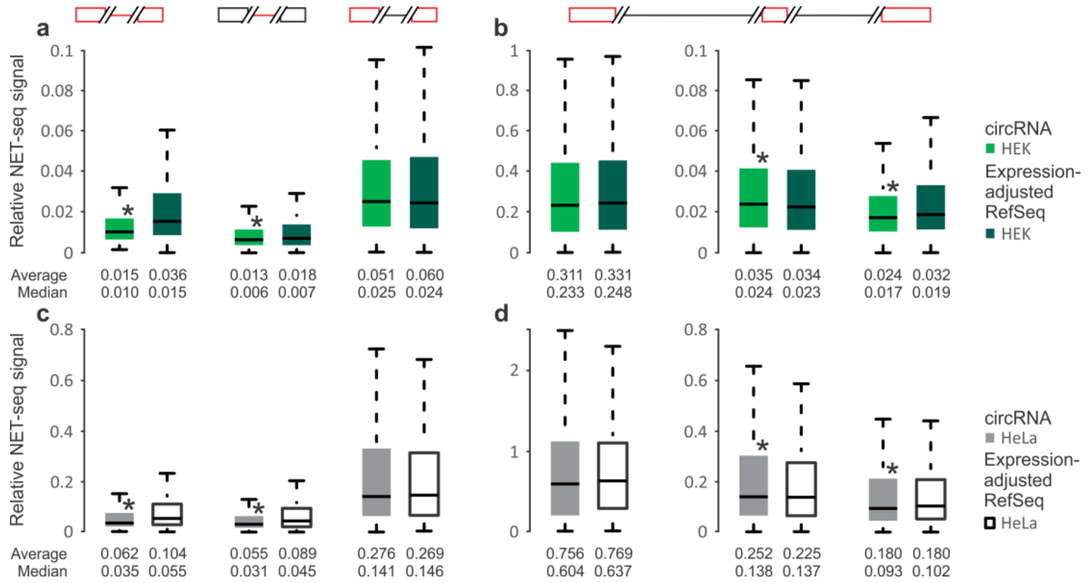

Supplementary Figure S9. NET-seq signal values (reflecting inverse Pol II speed across mRNA-coding genes) compared between circRNA-producing genes in HEK 293 (a,b) or HeLa (c,d) cells[53] and the corresponding expression-adjusted RefSeq genes (see 'Custom reference datasets' subsection in the Methods). (a,c) NET-seq signal measured across entre genes (left), all introns (middle) and all exons (right). (b,d) NET-seq signal measured for all first exons (left), all internal exons (middle) and all last exons (right). Asterisks denote significantly different NET-seq signal across different regions between the circRNA-producing genes and expression-adjusted genes (P-values <0.01, Mann-Whitney U test).

Supplementary Table S5. NET-seq signal values (Pol II/nt)[a] in the different regions of circRNA-producing genes compared to expression-adjusted RefSeq genes[b]. [a]Average (top) and median (bottom) values. [b]CircRNA-producing genes (623 genes in HEK, 550 in HeLa); expression-adjusted genes (7,355 in HEK, 6,267 in HeLa) (see 'Custom reference datasets' subsection of the Methods for details). [c]Faded-out numbers show P-values of Mann-Whitney U test between the circRNAs and expression-adjusted genes.

	Genes[b]	Entire gene[c]		Exons: All		Exons: First		Exons: Internal		Exons: Last		Introns	
HEK	RefSeq	0.0357<br>0.0152		0.0601<br>0.0242		0.3312<br>0.2482		0.0344<br>0.0226		0.0324<br>0.0188		0.0180<br>0.0069	
HEK	CircRNAs	0.0147<br>0.0102	< 2.2E-16	0.0513<br>0.0249	2.5E-1	0.3107<br>0.2325	5.7E-2	0.0351<br>0.0240	< 2.2E-16	0.0244<br>0.0171	4.7E-8	0.0132<br>0.0064	< 2.2E-16
HeLa	RefSeq	0.1044<br>0.0547		0.2685<br>0.1462		0.7688<br>0.6365		0.2246<br>0.13727		0.1799<br>0.1024		0.0894<br>0.0446	
HeLa	CircRNAs	0.0619<br>0.0354	< 2.2E-16	0.2762<br>0.1414	1.8E-1	0.7558<br>0.6044	2.4E-2	0.2515<br>0.1376	3.2E-5	0.1797<br>0.0928	6.1E-3	0.0554<br>0.0313	3.6E-10

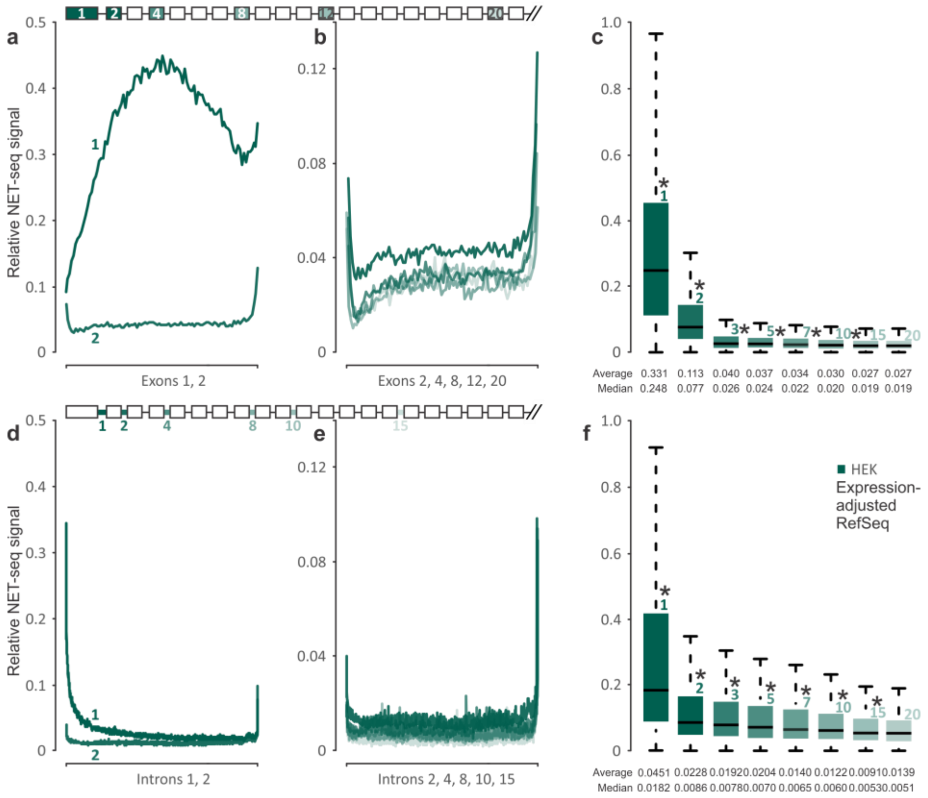

Supplementary Figure S10. NET-seq values of HEK 293 cells[53] along exons (a-c) and introns (d-f) in different ordinal positions of RefSeq genes. (c,f) Asterisks denote significantly different NET-seq signal between the indicated position and the subsequent position (*e.g.* between exon 1 and exon 2) (P-values < 0.01, Mann-Whitney U test).

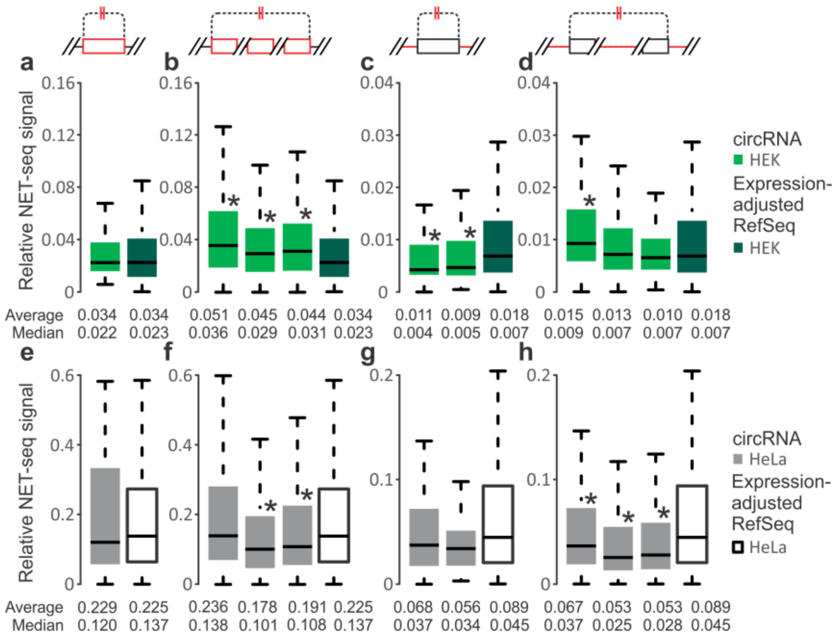

Supplementary Figure S11. NET-seq signal values (reflecting inverse Pol II speed across mRNA-coding genes) compared between circRNAs of HEK 293 (a-d) or HeLa (e-h) cells[53] and the corresponding expression-adjusted genes. NET-seq signal measured (a, e) between single-exon circRNA exons and internal RefSeq exons; (b, f) from right to left, multi-exon circRNA acceptor, internal, donor exons and internal RefSeq exons; (c, g) introns upstream (left) and downstream (middle) of single-exon circRNAs as well as RefSeq introns (right); (d, h) from left to right, introns upstream of, internal to, and downstream of multi-exon circRNAs and RefSeq introns. Asterisks denote significantly different NET-seq signal across different regions between the circRNAs and expression-adjusted genes (P-values <0.01, Mann-Whitney U test).

Discussion

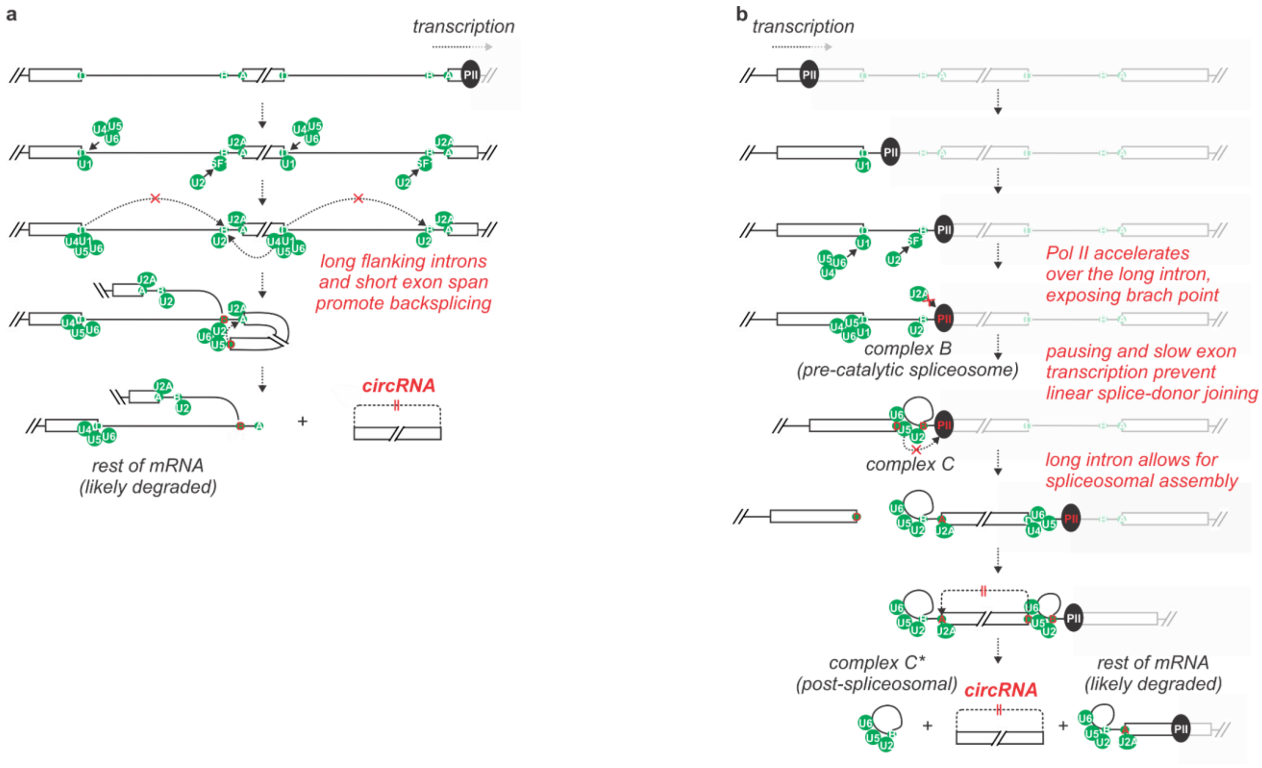

Supplementary Figure S12. Schematic of possible splicing-mediated mechanisms resulting in the synthesis of exonic circRNA and accounting for circRNA-specific features of gene structure and dynamics of transcription. (a) Model of post-transcriptional back-splicing where Pol II (PII; black oval) does not have an influence on the order of how exons and introns are presented to the splicing machinery. Note that among longer flanking introns (which might slow down their linear splicing) and short exon span of circRNAs (which might sterically favour back-splicing due to the proximity of back-spliced donor and acceptor), overall faster transcription of circRNA-producing genes may favour post-transcriptional back-splicing. (b) Model of co-transcriptional back-splicing where Pol II progression along the gene dictates sequence of splicing events and availability of the splice sites. In this case, features of transcriptional dynamics typical to circRNA-producing genes, such as the higher acceleration over acceptor intron, longer pausing at the intron-exon boundaries and larger Pol II speed difference between introns and exons may additionally facilitate back-splicing.

Supplementary Information References

14. Memczak, S. *et al.* Circular RNAs are a large class of animal RNAs with regulatory potency. *Nature* **495**, 333-338, doi:10.1038/nature11928 (2013).

53. Mayer, A. *et al.* Native elongating transcript sequencing reveals human transcriptional activity at nucleotide resolution. *Cell* **161**, 541-554, doi:10.1016/j.cell.2015.03.010 (2015).

58. Jakobi, T., Czaja-Hasse, L. F., Reinhardt, R. & Dieterich, C. Profiling and Validation of the Circular RNA Repertoire in Adult Murine Hearts. *Genomics Proteomics Bioinformatics* **14**, 216-223, doi:10.1016/j.gpb.2016.02.003 (2016).

59. Werfel, S. *et al.* Characterization of circular RNAs in human, mouse and rat hearts. *J Mol Cell Cardiol* **98**, 103-107, doi:10.1016/j.yjmcc.2016.07.007 (2016).

61. Yang, Y. C. *et al.* CLIPdb: a CLIP-seq database for protein-RNA interactions. *BMC Genomics* **16**, 51, doi:10.1186/s12864-015-1273-2 (2015).

62. Li, J. H., Liu, S., Zhou, H., Qu, L. H. & Yang, J. H. starBase v2.0: decoding miRNA-ceRNA, miRNA-ncRNA and protein-RNA interaction networks from large-scale CLIP-Seq data. *NAR* **42**, D92-97, doi:10.1093/nar/gkt1248 (2014).

90. Kozak, M. Context effects and inefficient initiation at non-AUG codons in eucaryotic cell-free translation systems. *Mol Cell Biol* **9**, 5073-5080 (1989).

91. Kozak, M. Recognition of AUG and alternative initiator codons is augmented by G in position +4 but is not generally affected by the nucleotides in positions +5 and +6. *Embo J* **16**, 2482-2492 (1997).

92. Kochetov, A. V. AUG codons at the beginning of protein coding sequences are frequent in eukaryotic mRNAs with a suboptimal start codon context. *Bioinformatics* **21**, 837-840, doi:bti136 doi:10.1093/bioinformatics/bti136 (2005).
